# Inferring Aggregated Functional Traits from Metagenomic Data Using Constrained Non-negative Matrix Factorization: Application to Fiber Degradation in the Human Gut Microbiota

**DOI:** 10.1371/journal.pcbi.1005252

**Published:** 2016-12-16

**Authors:** Sébastien Raguideau, Sandra Plancade, Nicolas Pons, Marion Leclerc, Béatrice Laroche

**Affiliations:** 1 Institut Micalis, INRA, AgroParisTech, Université Paris-Saclay, Jouy-en-Josas, France; 2 MaIAGE, INRA, Université Paris-Saclay, Jouy-en-Josas, France; 3 MGP, INRA, Université Paris-Saclay, Jouy-en-Josas, France; The Pennsylvania State University, UNITED STATES

## Abstract

Whole Genome Shotgun (WGS) metagenomics is increasingly used to study the structure and functions of complex microbial ecosystems, both from the taxonomic and functional point of view. Gene inventories of otherwise uncultured microbial communities make the direct functional profiling of microbial communities possible. The concept of community aggregated trait has been adapted from environmental and plant functional ecology to the framework of microbial ecology. Community aggregated traits are quantified from WGS data by computing the abundance of relevant marker genes. They can be used to study key processes at the ecosystem level and correlate environmental factors and ecosystem functions. In this paper we propose a novel model based approach to infer combinations of aggregated traits characterizing specific ecosystemic metabolic processes. We formulate a model of these Combined Aggregated Functional Traits (CAFTs) accounting for a hierarchical structure of genes, which are associated on microbial genomes, further linked at the ecosystem level by complex co-occurrences or interactions. The model is completed with constraints specifically designed to exploit available genomic information, in order to favor biologically relevant CAFTs. The CAFTs structure, as well as their intensity in the ecosystem, is obtained by solving a constrained Non-negative Matrix Factorization (NMF) problem. We developed a multicriteria selection procedure for the number of CAFTs. We illustrated our method on the modelling of ecosystemic functional traits of fiber degradation by the human gut microbiota. We used 1408 samples of gene abundances from several high-throughput sequencing projects and found that four CAFTs only were needed to represent the fiber degradation potential. This data reduction highlighted biologically consistent functional patterns while providing a high quality preservation of the original data. Our method is generic and can be applied to other metabolic processes in the gut or in other ecosystems.

## Author Summary

Microbial communities are highly complex ecosystems, harboring a high taxonomic diversity, rapidly varying in time. Focusing on the functional traits of microorganisms instead of species offers an interesting and complementary insight since quite often, owing to environmental selection pressure, traits are less variable in time and shared by many species. By providing gene inventories of otherwise uncultured microbial communities, Whole Genome Shotgun (WGS) metagenomics made the functional profiling of microbial communities possible, since community aggregated traits can be quantified by the abundance of relevant marker genes. Considering a global metabolic process in an ecosystem, we propose a method that exploits gene abundance data to determine combinations of functional traits characterizing this process. When applied to fiber degradation in the human gut microbiota, the method shows that only four complex functional traits are needed to characterize this process. The approach is generic and could be applied to other processes or ecosystems. It allows summarizing complex datasets by a limited number of biologically interpretable functional patterns.

## Introduction

Whole Genome Shotgun (WGS) metagenomics is increasingly used to study the structure and functions of complex microbial ecosystems. Thanks to a huge research effort, these high-throughput approaches are constantly improving and have already provided valuable information.

Recent achievements [1–3] for analyzing metagenomic reads have focused on genome or species reconstruction. They constitute an alternative to amplicon sequencing approaches such as 16S rDNA and provide potentially more robust tools for community structure assessment.

As a complement to these taxonomy oriented approaches, WGS metagenomics allows the direct functional profiling of microbial communities through gene inventories of otherwise uncultured microbial communities. The functional annotation of these genes, as well as the evaluation of their abundances requires the mapping of metagenomic reads on one or several functional databases which may include annotated, de novo assembled gene catalogs. It is a challenging task for which bioinformatic tools are still being developed (see [4] for a very recent review).

As early as 2005, Tringe et al. [5] demonstrated that functional binning could discriminate environments and determine the functional potential of microbial communities. Since then, several other groups used functional data to shed light on the processes taking place in the ecosystem, even in the absence of additional expression data (see e.g. [6, 7]), and developed frameworks to functionally characterize and compare ecosystems [4, 8]. Investigating complex microbial communities from a purely functional point of view provides an interesting insight because their taxonomic diversity may be very high and varying in time while, owing to environmental selection pressure, their ecosystemic functions are often ubiquitous and much more stable in time. The functional profiling of microbial communities has motivated the adaptation of concepts borrowed from environmental and plant functional ecology to the framework of microbial ecology, such as community aggregated traits [9, 10], which can be quantified through abundance computation of specific genes or predefined pathways from WGS data. These taxon-free approaches allow the study of key processes at the ecosystem level and correlate environmental factors and ecosystem functions [11].

Choosing relevant descriptors for ecosystem functions raises several issues. Using arbitrarily predefined pathways helps simplifying the systems description by creating combined descriptors, however it may not reflect all the information in the data. On the other hand, unsupervised statistical analyses such as e.g. principal component analysis (PCA) are quite often used to extract low-dimensional description of these high-dimensional datasets. However, they do not exploit all the richness of available information about biological processes at the individual or collective level, and may lead to irrelevant or hardly interpretable results. To overcome these issues, we propose a novel model based approach, mixing satistical data analysis and system biology. We focus on specific ecosystemic metabolic processes, described by a possibly high number of genes or functional markers abundances. Our aim is to infer a limited number of combined functional traits characterizing these processes, as well as their intensity in the ecosystem.

Our approach relies on Non-negative Matrix Factorization (NMF), a popular machine learning technique in data and image analysis. NMF, together with PCA belongs to a wide family of data analysis methods designed to solve blind source separation problems. These questions are widely encountered and cover all situations where a mixture of several signals originating from different unknown sources is observed (here gene abundances in samples) and one wishes to separate them and identify the sources (the combined functional traits) and the mixture coefficients.

NMF was previously used for genomic data mining in the context of microarray data analysis [12, 13]. More recently, it was introduced in the context of metagenomics for reads binning [14], or analyzing datasets from various ecosystems. In particular Jiang and co-authors used this approach as a “soft” clustering tool in different ecosystems. They studied pathway abundances in diverse environmental ecosystems [15], compared habitats in marine ecosystems using protein families profiles [16] and human body sites using phylogenetic and functional data [17].

In [18] the idea of coupling source separation analyses with prior knowledge was developed in the context of bipartite network reconstruction, using prior knowledge on the network structure, and applied to the reconstruction of regulatory signals from microarray data.

The originality of our work lies in the design of a constrained NMF approach where the constraints aim at selecting biologically relevant combined functional traits (the sources) to describe processes at the ecosystem level. The constraints are derived from available prior knowledge in a Bayesian perspective. Moreover, we propose a careful multicriteria selection procedure to select the relevant number of combined functional traits.

As a proof of concept, we applied our approach to the modelling of ecosystemic functional traits of fiber degradation by the human gut microbiota based on 1408 samples of gene abundances.

## Results

### A model of combined aggregated functional traits

Quantitative metagenomics allows the study of metabolic processes at the ecosystem level, by producing metagenes abundances with functional annotation. Taking advantage of this information, we consider a metabolic process occurring in an ecosystem. We assume that this process is described by a list of biochemical reactions, each associated to what we call a *functional marker*. A functional marker is a group of genes, or modular elements in genes, able to control the production of enzymes involved in a reaction. Typical functional markers are Kegg Orthologies (KO). The abundance of a functional marker is defined as the sum of the abundances of all the metagenes identified as belonging to the group.

Our first modelling hypothesis is the existence of patterns underlying functional markers abundances in the ecosystem. Indeed, within a microbial ecosystem, genes can be looked at in a hierarchical fashion. They are first associated on microbial genomes, which are further grouped at the ecosystem level into subcommunities of microorganisms. These subcommunities involve hundreds of different bacterial species linked by complex co-occurrences or interactions. Moreover they are influenced by environmental factors (e.g. nutrients or temperature). The set of functional marker abundances within each of these subcommunities form a characteristic pattern in the ecosystem.

**Fig 1:**
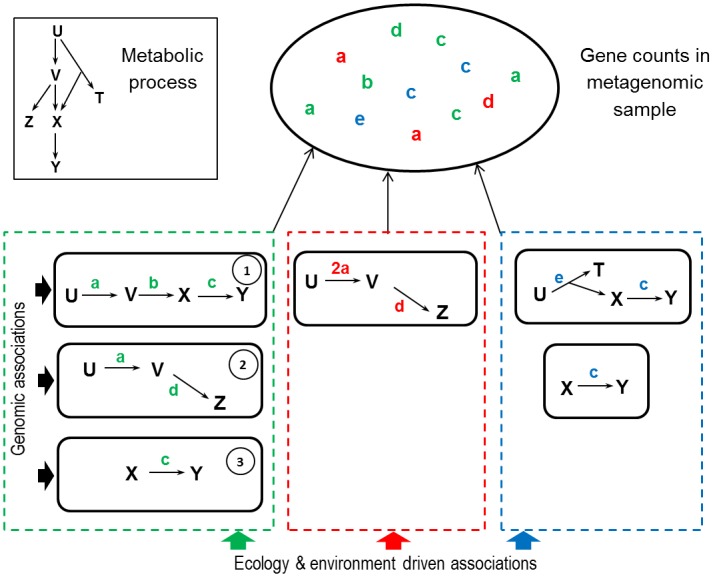
Hierarchical structure underlying metagenomic data. We consider a metabolic process in a microbial ecosystem involving a substrate U, metabolites V, X, Y, Z, T and the set of reactions U → V, V → X, X → Y, V → Z and U → T + X, respectively catalyzed by proteins synthesized by genes in KEGG Orthology (KO) groups **a**, **b**, **c**, **d** and **e**. Gene counts stem from an underlying hierarchical organization. Genes are associated within bacterial genomes (solid black lines) and through ecosystem level association patterns (green, red and blue ticked lines). In this example, the green and blue boxes can be interpreted as trophic chains corresponding to two distinct pathways for substrate degradation. The red box can be interpreted as an alternative to the green one, involving different bacterial groups, depending on the host diet or life history. Note that the red box involves (possibly several) species harbouring two copies of gene **a** in their genomes.

This hypothesis is illustrated in the simplistic example of metabolic process in Fig 1. Here, the observed functional marker abundances originate from three subcommunities, resulting in three patterns. The ecological interpretation of the first pattern (green box) could be a trophic chain in which metabolite *X* is produced by bacterial species of type 1 and used by bacterial species of type 3 as well as an association between bacterial species of type 1 and 2 for other ecological reasons which cannot be elucidated with the observation of this particular metabolic process only. Therefore functional marker abundances in samples should reflect this structure.

**Fig 2:**
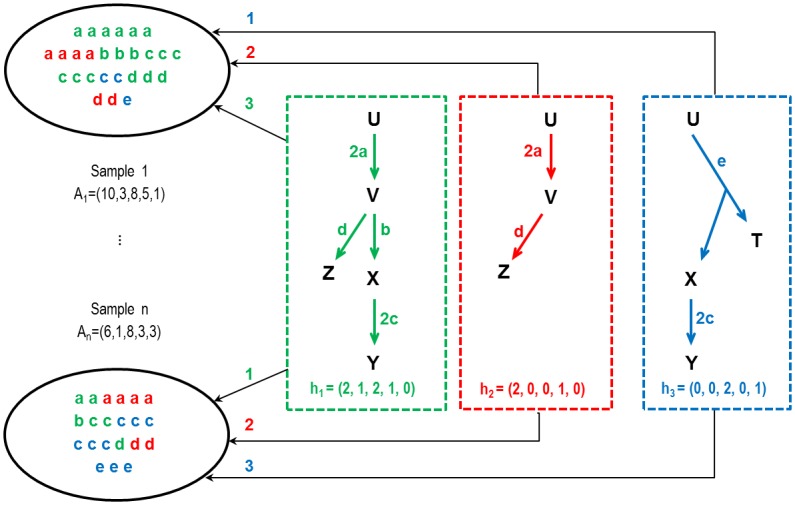
Combined Aggregated Functional Traits. The gene association patterns in Fig 1 correspond to stable structures, representing different ways to fulfill the metabolic function of interest at the ecosystem level. At the bottom of each box, vectors *h*_1_, *h*_2_ and *h*_3_ represent functional markers abundances. We call these vectors Combined Aggregated Functional Traits (CAFTs). They should be found in all samples, possibly in varying proportions. Sample 1 is decomposed as *A*_1_ = 3*h*_1_ + 2*h*_2_ + *h*_3_ and sample *n* as *A*_*n*_ = *h*_1_ + 2*h*_2_ + 3*h*_3_.

Our second modelling hypothesis is motivated by the fact that even if in many microbial communities microbial species composition varies over time and samples, metabolic processes at the community level are ubiquitous and much more stable in time. Consequently, we assume that the patterns mentioned above are shared by all samples of the ecosystem, in variable proportions because they were selected by specific environmental constraints. For instance, for the gut ecosystem these include anaerobic conditions, temperature or digestion residue composition coming from the host diet. As illustrated on Fig 2, when considering the abundances of functional markers in several samples, each of these patterns can be characterized by a line vector of functional marker frequencies, which we define to be a Combined Aggregated Functional Trait (CAFT), in the meaning that it represents a combination of quantifiable characteristics (functional markers) of aggregated microbial communities.

Following our hypotheses, functional marker abundances in each sample are modelled as mixtures of underlying CAFTs. For each sample *i* = 1, …, *n* and marker *j* = 1, …, *r*, the observed frequency *a*_*ij*_ of marker *j* in sample *i* is the sum of the contributions of all CAFTs. This leads to

$$a_{ij}≃\sum_{l=1}^{k}w_{il}h_{lj}$$ (1)

where *h*_*l*_ = (*h*_*l*1_, …, *h*_*lr*_) is the characterization in terms of *r* markers frequencies of the *l*^*th*^ CAFT and *w*_*i*_ = (*w*_*i*1_, …, *w*_*ik*_) is the vector of the intensities or weights of the *k* CAFTs in sample *i*.

Note that each *h*_*l*_ is defined up to a multiplication by a positive constant, provided that its weights in the samples are scaled accordingly. The *ℓ*^1^-scaled version *h*_*l*_/(∑_*j*_ *h*_*lj*_) can be interpreted as the vector of relative frequencies of functional markers within the subcommunity characterized by trait *l*.

In the rest of the paper, we denote the (*n* × *r*) data matrix by *A*, the (*k* × *r*) matrix whose lines are the *h*_*l*_ by *H* and the (*n* × *k*) matrix whose lines are the *w*_*i*_ by *W*. *H* will be called the trait matrix and *W* the weight matrix.

### Constraints

By definition the entries of the weight and trait matrices *W* and *H* are assumed to be non-negative, we denote this condition by

W≥0,H≥0. (2)

We now show how prior knowledge on microbial genomics and metabolism can be exploited in order to select biologically relevant CAFTs.

A first important consequence of our modeling framework is that genomic information can be translated into constraints on CAFTs structure. Indeed, by construction, the CAFT *h*_*l*_ characterizes a gene pattern associated in each sample *i* to a subcommunity *C*_*il*_. Therefore, in each of these subcommunities, the total abundance of any functional marker *j* is proportional to *h*_*lj*_. Thus, denoting *n*_*xj*_ the number of functional markers *j* in the genome of a microorganism *x* in the ecosystem, it follows that

$$\sum_{x\in C_{il}}n_{xj}=w_{il}h_{lj}.$$ (3)

From Eq (3) we observe that any constraint on the structure of the genomes (the *n*_*xj*_) satisfied by all *x* in the ecosystem induces a constraint on the CAFTs (the *h*_*lj*_).

As an example, let us assume that two markers *j* and *j*′ are such that for each microorganism in the ecosystem, if *j*′ is not present in its genome, then neither is *j*. This implies that for each microorganism *x*, there is a non-negative constant *R*_*x*, *j*, *j*′_ satisfying

$$n_{xj}=R_{x,j,j^{\prime }}n_{xj^{\prime }},$$ (4)

where we arbitrarily set *R*_*x*, *j*, *j*′_ = 0 when *n*_*xj*_ = *n*_*xj*′_ = 0. Then as a consequence of Eq (3) we have for each CAFT *l*

$$h_{lj}\leq \delta h_{lj^{\prime }},$$ (5)

where *δ* = sup_*x* ∈ Ecosystem_ *R*_*x*, *j*, *j*′_.

In the absence of any other information, exploiting genomic information only could lead to a large number of constraints. For instance, constraining the ratio of abundances in pairs of markers as above requires assessing the existence of constants such that Eq (4) holds for *r*(*r* − 1) ordered pairs, for each genome *x*. This is why we propose to combine genomic information with metabolic information, when possible, in order to select a limited but relevant set of constraints on the CAFTs.

In order to do this, we formulate two additional assumptions. First, we assume that a subset of reactions in the metabolic process under consideration are well known, so that a list of metabolites that are consumed or produced by each reaction can be extracted.

Second, we assume that metabolites are partitioned in two classes. The first one corresponds to metabolites that are known to be exported out of the cell, from experimental evidence or published data. The second one gathers metabolites that are actually known to stay within microbial cells, as well as metabolites for which this is strongly suspected. For convenience, these two classes will be called the extracellular and intracellular metabolites, although this may be misleading in some cases.

Following similar steps as in eqs (3–5), a subset *M*_1_ of the class of intracellular metabolites can be derived from genomic information on the microorganisms present in the ecosystem, as well as a positive constant *δ*_1,*m*_ for each *m* in *M*_1_, such that in each CAFT *h*_*l*_ the marker frequencies associated to the production or consumption of *m* should satisfy

$$\sum_{\text{j}\;\text{producing}\;\text{m}}h_{lj}\leq \delta_{1,m}\sum_{\text{j'}\;\text{consuming}\;\text{m}}h_{lj^{\prime }}$$ (6)

In the same way, there is a subset *M*_2_ of intracellular metabolites such that for each *m* in *M*_2_, each CAFT *h*_*l*_ should satisfy

$$\sum_{\text{j'}\;\text{consuming}\;\text{m}}h_{lj^{\prime }}\leq \delta_{2,m}\sum_{\text{j}\;\text{producing}\;\text{m}}h_{lj}$$ (7)

The intuitive idea behind such constraints is the following. Assuming that functional markers are relevant proxies of metabolic functions, it could be expected that if a genuinely intracellular metabolite is produced in the subcommunity characterized by a given CAFT it has to be used. This inspires constraint (6), since it imposes that the number of markers associated to reactions that produce a given intracellular metabolite is zero if the number of markers associated to reactions that consume the same intracellular metabolite is zero. Conversely, it could be expected that a metabolite cannot be used if it is not produced, which is enforced by constraint (7). This justifies the absence of constraints imposed on functional markers associated to reactions consuming or producing for the class of extracellular metabolites as the latter can actually be imported or exported outside bacterial cells in the ecosystem.

However, the set of metabolites and reactions under consideration is defined in the context of a specific metabolic process, given the available knowledge. Thus it may be that in this process, some intracellular metabolites accumulate in the cells. It may also be that the selected reactions ignore metabolic routes, because they are unknown or correspond to other metabolic processes, that could produce or use the metabolites, and finally it may also happen that we were wrong in assuming that some poorly studied metabolites are intracellular. Consequently, depending on metabolites, either none, one or both constraints (6) and (7) may be satisfied. In the case where both constraints apply, the frequencies of markers associated to production and consumption are both either zero or positive with consumption over production marker frequency ratio lying in [1/*δ*_1,*m*_, *δ*_2,*m*_].

The theoretical construction of the two sets *M*_1_, *M*_2_ and the positive constants *δ*_1,*m*_ and *δ*_2,*m*_ is detailed in the Materials and Methods section, as well as their practical approximation from known bacterial genomes.

The set of constraints (6) and (7) can be expressed in matrix notation as

$$F_{\Delta }H^{T}\leq 0$$ (8)

where Δ is the set of parameters Δ = {*δ*_1,*m*_, *m* ∈ *M*_1_} ∪ {*δ*_2,*m*_, *m* ∈ *M*_2_} and *F*_Δ_ is a coefficient matrix depending on Δ.

Note that, although they include metabolic information and take the familiar form of linear inequality constraints (6) and (7) are constraints on gene frequencies in subcommunities, they are not constraints on metabolic fluxes.

### Inference of CAFT and weights

#### Nonnegative Matrix Factorization formulation

The determination of matrices *W* and *H* which satisfy Eqs (1) and (2) can be formulated as an approximate Nonnegative Matrix Factorization (NMF) problem (see e.g. [19]). The mathematical formulation of the problem requires first to choose a dissimilarity measure for the approximate factorization Eq (1). In our algorithm, we considered the Frobenius norm. Moreover, as functional markers may display different order of magnitude, a scaling per functional marker is operated by considering the *ℓ*^2^-columnwise normalization of *A*, denoted $\tilde{A}$. Therefore, the dissimilarity considered is

$$\|\tilde{A}-W\tilde{H}{\|}_{F}^{2}=\sum_{i=1}^{n}\sum_{j=1}^{r}{\left({\tilde{a}}_{ij}-\sum_{l=1}^{k}w_{il}{\tilde{h}}_{lj}\right)}^{2}$$ (9)

where $\tilde{H}$ is the scaled trait matrix. Matrix *H* is then recovered by multiplying each column of $\tilde{H}$ by the *ℓ*^2^-norm of the corresponding column in *A*.

The NMF algorithms which aim at minimizing this distance require the use of regularizing terms in order to lift identifiability problems and filter noisy data during the minimization process. We consider a regularizing term that promotes sparsity over the columns of $\tilde{H}$, which encourages markers to be present in a limited number of CAFTs (see Materials and Methods).

Finally, we impose the additional constraint (8). Let ${\tilde{F}}_{\Delta }$ be the scaled matrix *F*_Δ_ such that the constraint (8) is equivalent to ${\tilde{F}}_{\Delta }{\tilde{H}}^{T}\leq 0$. Therefore, the inference of the CAFTs and weights is obtained by solving the following minimization problem

$$\begin{array}{c} \min_{W,\tilde{H}}\;\|\tilde{A}-W\tilde{H}{\|}_{F}^{2}+{\alpha (\|W\|}_{F}^{2}+\|1^{T}\tilde{H}{\|}_{2}^{2})\\ W\geq 0,\;H\geq 0,\;{\tilde{F}}_{\Delta }{\tilde{H}}^{T}\leq 0 \end{array}$$ (10)

where 1 denotes the vector of length *k* whose entries are equal to 1.

It is well known that problems like Eq (10) may have multiple solutions or local minima. To circumvent this issue, similarly to most approaches presented in the literature, our algorithm is repeatedly implemented with random initializations, and the one providing the best reconstruction of the data is chosen. More details are given in the Inference section in Materials and Methods.

#### Selection of parameters

The minimization problem Eq (10) requires to choose a value for the number of CAFTs *k* and the tuning parameter *α*. Parameter selection procedures proposed in most applications of NMF to biological data are based upon stability of the weight matrix *W* over several initializations of the iterative algorithm used to solve the problem [12, 13, 15, 16], in particular regarding the clustering of samples. Since our use of NMF focuses on extracting reproducible biological mechanisms characterized by the trait matrix *H*, we are more interested in biological stability than numerical one. Therefore, we propose an adaptation of the concordance index developed by [16], which measures the concordance between two matrices of profiles, to evaluate the concordance of the CAFTs computed on independent data sets. In our approach, the reproducibility of *H* on a new data set is mimicked by repeatedly splitting the set of biological samples, performing the NMF decomposition on each subset and evaluating the similarity between the two trait matrices via the concordance index. Even if the index formula is identical to the one proposed by [16], the interpretation differs since the authors assessed the reproducibility between various initializations of the NMF algorithm implemented on the whole data set. Note that, as mentioned in the previous section, our algorithm includes repeated random initializations as well, and each NMF decomposition computed on a subset of the data is obtained by chosing the initialization which offers the best reconstruction. To enhance interpretation, this criterion is associated with two more classical ones: change of slope of the reconstruction error and bi-cross-validation error [20]. The selected value is the one which optimizes the concordance of *H* while ensuring that the other criteria are acceptable. More details are given in Materials and Methods.

### Impact of the constraint: Toy model

**Fig 3:**
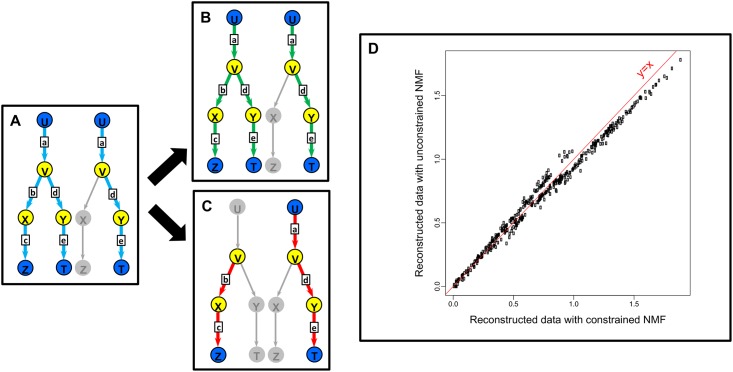
A toy example to illustrate the impact of the constraints. We consider a metabolic process in a microbial ecosystem, which involves five reactions (arrows) associated to functional markers **a**, **b**, **c**, **d** and **e**, three extracellular metabolites *U*, *Z* and *T* (blue circles) and three intracellular metabolites *V*, *X* and *Y* (yellow circles). The metabolic process is organized according two CAFTs, displayed in box A. The CAFTs were inferred from 80 samples of functional markers counts, generated from random mixtures of the two CAFTs. The estimated CAFTs are shown, both for the constrained (box B) and unconstrained NMF (box C). Box D shows the reconstructed counts in each sample (*WH*)_*ij*_ with constrained (x-axis) and non-constrained (y-axis) NMF.

The constrained NMF algorithm as well as an unconstrained version were implemented on a toy model in order to illustrate the improvement in terms of CAFT analysis brought by the constraints. We considered a microbial ecosystem in which a metabolic process characterized by a simple 5 reactions graph takes place, each reaction being associated to a functional marker. The metabolic process was assumed to be structured in two CAFTs. The first CAFT characterizes a subcommunity in the ecosystem in which all the functional markers are present in equal proportion, while the second CAFT characterizes a subcommunity in which only three functional markers are present, in equal proportion. The metabolic graph and the true CAFTs are displayed in Fig 3A. Thus, the true CAFT composition matrix was taken equal to

$$\bar{H}=\left(\begin{array}{c} 1\;1\;1\;1\;1\\ 1\;0\;0\;1\;1 \end{array}\right)$$

We built a matrix *A* for 80 samples assumed to be mixtures of CAFT 1 and CAFT 2. We first generated a matrix $\bar{W}$ of abundances of CAFT 1 and CAFT 2 for the 80 samples, with entries randomly drawn from a uniform distribution, and computed the corresponding marker count matrix $B=\bar{W}\bar{H}$. Noisy counts were generated from *B* according to the formula

$$a_{ij}=b_{ij}\left(1+0.2\epsilon_{ij}\right)$$

where the *ϵ*_*ij*_ are independent, centered Gaussian variables with unit variance.

In order to exemplify the construction of the constraints, we assumed that a set of three highly prevalent bacteria in this ecosystem was known. Bacteria are represented by the vectors *x*_1_, *x*_2_ and *x*_3_ of functional markers counts in their genomes

$$\begin{array}{c} x_{1}=\left(1,\;1,\;1,\;0,\;0\right)\\ x_{2}=\left(1,\;0,\;0,\;1,\;0\right)\\ x_{3}=\left(0,\;0,\;0,\;0,\;1\right) \end{array}$$

We assumed that based on published data on the physiology of these bacteria and possibly other bacteria in the ecosystem that are less prevalent, we knew that metabolites *U*, *Z* and *T* were extracellular. For instance, these data may come from *in vitro* culture experiments. We also assumed that the metabolites *V*, *X* and *Y* were not detected in the extracellular medium in these type of experiments, and considered them as intracellular by default.

We used these representative genomes, together with the metabolic graph of Fig 3A and applied the rules described in the Materials and Methods section to build a set of constraints on the CAFTs. Considering metabolite *V*, it is known to be produced by the reaction associated to marker *a* and consumed by the reactions associated to *b* and *d*. The inspection of the three genomes led to

$$\begin{array}{c} n_{1a}=1=1\times \left(n_{1b}+n_{1d}\right)\;\text{in}\;x_{1}\text{,}\;\text{since}\;n_{1b}+n_{1d}=1\\ n_{2a}=1=1\times \left(n_{2b}+n_{2d}\right)\;\text{in}\;x_{2}\text{,}\;\text{since}\;n_{2b}+n_{2d}=1\\ n_{3a}=0=0\times \left(n_{3b}+n_{3d}\right)\;\text{in}\;x_{3}\text{,}\;\text{since}\;n_{3b}+n_{3d}=0 \end{array}$$

Therefore Eq (11) is satisfied on the three reference genomes. Thus, *V* belongs to ${\tilde{M}}_{1}$ with *δ*_1*V*_ = max(1, 1, 0) = 1. As described in the Materials and Methods section, we compensated for calibrating *δ*_1*V*_ on a limited number of genomes instead of all the genomes in the ecosystem by applying a multiplicative factor of 2, which seems sensible here since the highly prevalent genome have at most one copy of each functional marker in their genome. We obtained the following constraints

$$h_{l1}\leq 2\left(h_{l2}+h_{l4}\right)$$

Similarly, *X* belongs to ${\tilde{M}}_{1}$ with *h*_*l*2_ ≤ 2*h*_*l*3_. However, *Y* is not in ${\tilde{M}}_{1}$ as Eq (11) is not satisfied for genome *x*_2_, here 33% of the reference genomes. In the same way, *V* and *X* belong to ${\tilde{M}}_{2}$ with *h*_*l*2_+*h*_*l*4_ ≤ 2*h*_*l*1_ and *h*_*l*3_ ≤ 2*h*_*l*2_, and *Y* is discarded.

We compared the CAFTs reconstructed using the constrained (Fig 3B) and unconstrained (Fig 3C) NMF inference. The NMF inference with constraints recovers the true CAFTs, while the unconstrained inference fails to provide relevant biological results. Besides, both constrained and unconstrained NMF provide a similar reconstruction of the data, as shown in Fig 3D. Indeed, from a mathematical point of view, the reconstructed CAFTs with and without constraints span the same linear space.

Therefore, while both constrained and unconstrained NMF provide usable results for biological sample analysis, only the constrained version is relevant for CAFT inference.

### Application: Fiber catabolism CAFT in the human gut microbiota

#### Data and metabolic process description

**Fig 4:**
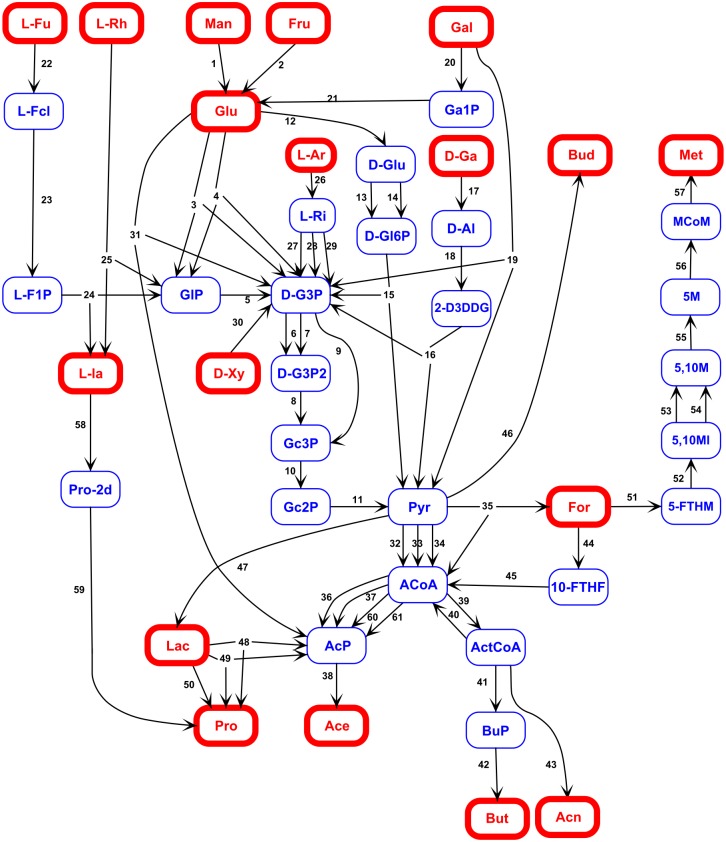
Reaction graph of the biological catabolic pathway from simple sugars to SCFA and methane. The nodes of the graph are the 43 selected metabolites, red nodes correspond to the 18 extracellular metabolites and blue nodes are the 25 extracellular, their full names and status are given in Table 3. The 61 edges of the graph correspond to the 61 selected functional markers listed in Table 2 with their associated reaction. More details can be found in S1 Table.

We exploited gene frequencies in 1408 Whole Genome Shotgun metagenomic samples from 8 distinct studies [3, 21–27] focused on different health status and human populations (Europe, China, USA). For each sample, gene frequencies were obtained by counting sample reads over the Integrated Genome Catalog (IGC) [21] composed of 9.9 million non redundant genes. Functional markers of fiber catabolism were defined from Kegg Orthologies (KO) [28, 29] as well as Glycosides Hydrolases (GH) and Pectin Lyases (PL) families [30]. A total of 86 relevant markers (25 GH-PL and 61 KO, listed in Tables 1 and 2) were carefully manually selected as being specifically associated to fiber catabolism in the human gut microbiota. Finally, a 1408 × 86 matrix of marker frequencies *A* was derived by summing the corresponding gene frequencies. The graph representing sugar fermentation is displayed on Fig 4. It comprises 43 major metabolites, among which 25 are known or assumed to be intracellular and 18 are known to be extracellular. It is oriented according to the biological catabolic pathway from the hydrolysis of fiber leading to simple sugars subsequently fermented to Short Chain Fatty Acid (SCFA) and methane.

**Table 1:** List of the 25 selected GH/PL.

GH3	GH13	GH32	GH51	GH120
GH5	GH16	GH39	GH74	GH127
GH8	GH26	GH43	GH91	PL1
GH9	GH28	GH44	GH94	PL9
GH10	GH30	GH48	GH115	PL11

**Table 2:** List of the 61 functional markers of sugar catabolism.

Reaction	KO(s)	Reaction	KO(s)
1	K01809	32	K00169/170/171/172
2	K00882	33	K00627
3	K06859	34	K03737
4	K01810	35	K00656
5	K01803	36	K04020
6	K00150	37	K00625
7	K00134	38	K00925
8	K00927	39	K00626
9	K00131	40	K01034/K01035
10	K01834	41	K00634
11	K01689	42	K00929
12	K00036	43	K01574
13	K01057	44	K01938/K00288/K01491
14	K07404	45	K00297
15	K01690	46	K00004/K03366
16	K00874	47	K00016
17	K00041	48	K01847
18	K01685	49	K01848/K01849
19	K00883	50	K01026
20	K00849	51	K00672
21	K00965	52	K01499
22	K01818	53	K00319
23	K00879	54	K13942
24	K01628	55	K00320
25	K01813	56	K00577/578/…/584
26	K01804	57	K00399/K00401/K00402
27	K01786	58	K01699/K13919/K13920
28	K03077	59	K13922
29	K03080	60	K13788
30	K00854	61	K15024
31	K01621		

**Table 3:** List of the 43 selected metabolites.

Metabolite	Abbreviation	Status
Acetate	Ace	Extracellular
Acetone	Acn	Extracellular
Butanediol	Bud	Extracellular
Butanoate	But	Extracellular
D-galacturonate	D-Ga	Extracellular
D-Xylose	D-Xy	Extracellular
Formate	For	Extracellular
Fructose	Fru	Extracellular
Galactose	Gal	Extracellular
Glucose	Glu	Extracellular
Lactate	Lac	Extracellular
L-Arabinose	L-Ar	Extracellular
L-Fucose	L-Fu	Extracellular
L-Lactaldehyde	L-la	Extracellular
L-Rhamnose	L-Rh	Extracellular
Mannose	Man	Extracellular
Methane	Met	Extracellular
Propanoate	Pro	Extracellular
10-Formyltetrahydrofolate	10-FTHF	Intracellular
2-Dehydro-3-deoxy-D-gluconate	2-D3DDG	Intracellular
5,10-Methenyltetrahydromethanopterin	5,10Ml	Intracellular
5,10-Methylenetetrahydromethanopterin	5,10M	Intracellular
5-Formyl-5,6,7,8-tetrahydromethanopterin	5-FTHM	Intracellular
5-Methyl-5,6,7,8-tetrahydromethanopterin	5M	Intracellular
Acetoacetyl-CoA	ActCoA	Intracellular
Acetyl phosphate	AcP	Intracellular
Acetyl-CoA	ACoA	Intracellular
Butanoyl phosphate	BuP	Intracellular
D-Altronate	D-Al	Intracellular
D-Gluconate 6-phosphate	D-Gl6P	Intracellular
D-Glucono-1,5-lactone 6-phosphate	D-Glu	Intracellular
Dihydroxyacetone phosphate	GlP	Intracellular
Galactose 1-phosphate	Ga1P	Intracellular
Glyceraldehyde 3-phosphate	D-G3P	Intracellular
Glycerate 2-phosphate	Gc2P	Intracellular
Glycerate 3-phosphate	Gc3P	Intracellular
Glycerate1,3 di-phosphate	D-G3P2	Intracellular
L-Fuculose	L-Fcl	Intracellular
L-Fuculose 1-phosphate	L-F1P	Intracellular
L-Ribulose	L-Ri	Intracellular
Methyl-CoM	MCoM	Intracellular
Propane 1,2-diol	Pro-2d	Intracellular
Pyruvate	Pyr	Intracellular

For each of the 25 intracellular metabolites, the constraints were calibrated using 190 core gut microbial genomes (see Materials and Methods). Both constraints (6) and (7) were implemented together on the functional markers associated to the production and consumption of 17 intracellular metabolites, only one of them on 4 intracellular metabolites, resulting in 38 constraints. The functional markers associated to the production and consumption of the remainig 4 metabolites, namely D-Gluconate 6-phosphate, D-Glucono-1,5-lactone 6-phosphate, L-Fuculose 1-phosphate and Propane 1,2-diol were left unconstrained. The details of the constraints are given in Table 4. We assessed the sensitivity of the inference procedure to the thresholds used to define the constraints and observed a moderate sensitivity (see Materials and Methods for more details). The overall data generation process and the graph building procedure are detailed in section Materials and Methods.

**Table 4:** Constraints on the functional markers associated to the production and consumption of intracellular metabolites in the catabolic pathway from simple sugars to SCFA and methane.

Metabolite	*δ*_1,*m*_ in Eq (6)	*δ*_2,*m*_ in Eq (7)
10-Formyltetrahydrofolate	5	20
2-Dehydro-3-deoxy-D-gluconate	5	–
5,10-Methenyltetrahydromethanopterin	5	5
5,10-Methylenetetrahydromethanopterin	5	5
5-Formyl-5,6,7,8-tetrahydromethanopterin	5	5
Methyl-CoM	5	5
5-Methyl-5,6,7,8-tetrahydromethanopterin	5	5
Acetoacetyl-CoA	–	5
Acetyl-CoA	20	5
Acetyl phosphate	10	10
Butanoyl phosphate	5	5
D-Altronate	10	10
D-Gluconate 6-phosphate	–	–
Glycerate1,3 di-phosphate	5	5
Glyceraldehyde 3-phosphate	30	5
D-Glucono-1,5-lactone 6-phosphate	–	–
Galactose 1-phosphate	–	10
Glycerate 2-phosphate	15	5
Dihydroxyacetone phosphate	10	10
Glycerate 3-phosphate	10	15
L-Fuculose	5	5
L-Fuculose 1-phosphate	–	–
L-Ribulose	5	–
Propane 1,2-diol	–	–
Pyruvate	5	10

#### Parameter selection

**Fig 5:**
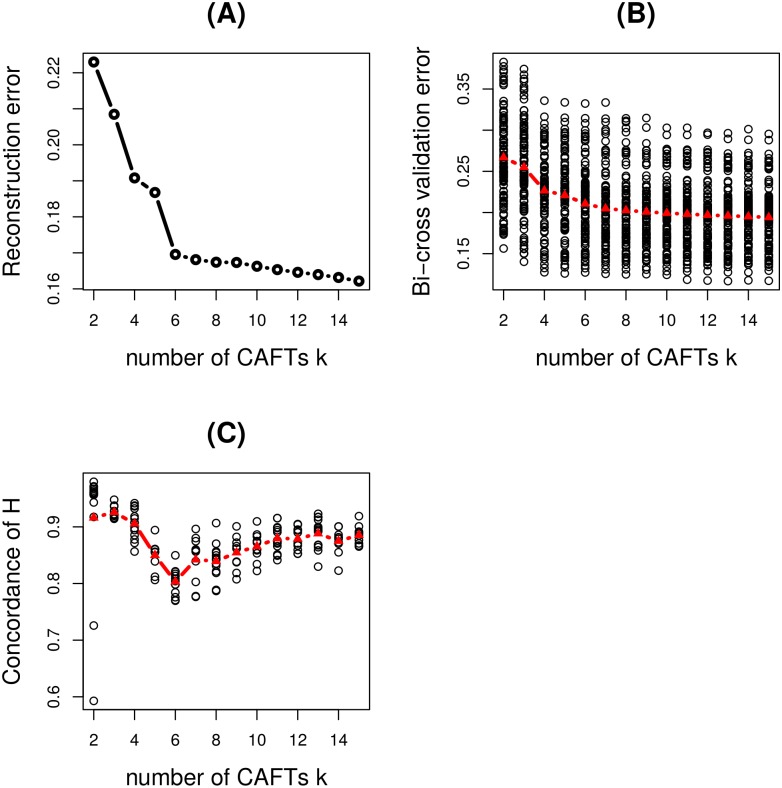
Fiber digestion in the human gut: selection of the number of CAFTs *k*. The profiles of the three criteria as a function of *k* for the selected value of *α* = 0.031 are displayed. **(A)** Reconstruction error; **(B)** 10-fold bi-cross validation error; **(C)** Concordance of *H* over repeated splits of the sets of samples. In **(B)** and **(C)** the value of the criterion for each split as well as the median are shown.

In order to select the optimal values of the parameters *α* and *k*, the concordance index between matrices *H* computed on 40 random splits of the data, the reconstruction error and the 10-fold bi-cross-validation error were computed on a grid of parameters, following the procedure presented in Material and Methods. We present here the selection of the number *k* of CAFTs, and the details for *α* are given in Supplementary. The profiles of the three criteria as a function of the regularization parameter *α* are displayed in S3 Text, for various numbers of CAFTs *k*; the value *α* = 0.031 was selected. Fig 5 displays the three criteria as a function of *k* for the selected value of *α*. The reconstruction error curve undergoes two changes of slope for *k* = 4 and 6. The profile of the concordance of *H* ensures a good reproducibility of the trait matrix *H* when less than 4 CAFTs are considered. Moreover, the bi-cross-validation error displays a significant decrease until 4 CAFTs, and a more moderate one for larger values of *k*; besides, the variations as *k* increases are globally weak with respect to the range of values over the splits. In the context of our analysis, in which the main focus is the inference of metabolic traits shared by all samples, the value *k* = 4 was chosen.

#### Analysis of the weight matrix W*

**Fig 6:**
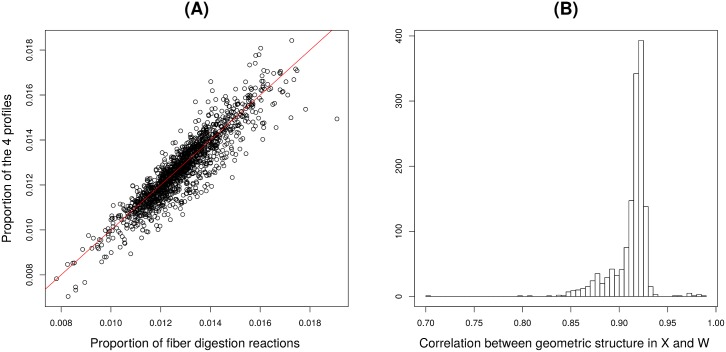
Fiber digestion in the human gut: analysis of W*. **(A)** For each biological sample, the total abundances in the 4 CAFTs (row sums of W* H*) is displayed as a function of the proportion of the 86 fiber digestion reactions in the metagenome (row sums of *A*). **(B)** For each sample, the vector of distances to the other samples is computed from the rows of *A* and of W* respectively; the histogram of Pearson correlations between theses two distance vectors for the 1408 biological samples is displayed.

The constrained NMF decomposition may be looked at as a dimension reduction analysis, where biological samples originally characterized by their abundances in 86 functional markers are described by their abundances in the 4 CAFTs displayed in the matrix W*. We checked that the information on biological samples carried out by *A* was well preserved in W*. First of all, the row sums of *A* quantify the proportion of genes devoted to the 86 fiber digestion reactions in each biological sample; they vary between 0.8% and 1.9% across the 1408 samples. Fig 6(A) shows that this proportion is highly correlated to the the total abundance in the 4 CAFTs characterized as the row sums of W* H* (Pearson correlation 0.91). Thus, the individual variability of the proportion of fiber digestion related genes is properly recovered by the NMF decomposition.

Moreover, the two-by-two distances between the rows of *A* characterize the geometric structure of the biological sample population with respect to fiber digestion mechanisms. A similar geometric structure is defined by the rows of W*. We compared the distances of each sample to the others, respectively in *A* and in W* with Pearson correlation. The histogram of these correlations for the 1408 samples points out that all correlations are larger than 0.7, and that 99% are larger than 0.85, which indicates that the similarities between biological samples is well preserved in the reduced spaced induced by the NMF decomposition (Fig 6(B)).

#### The CAFTs form coherent pathways

**Fig 7:**
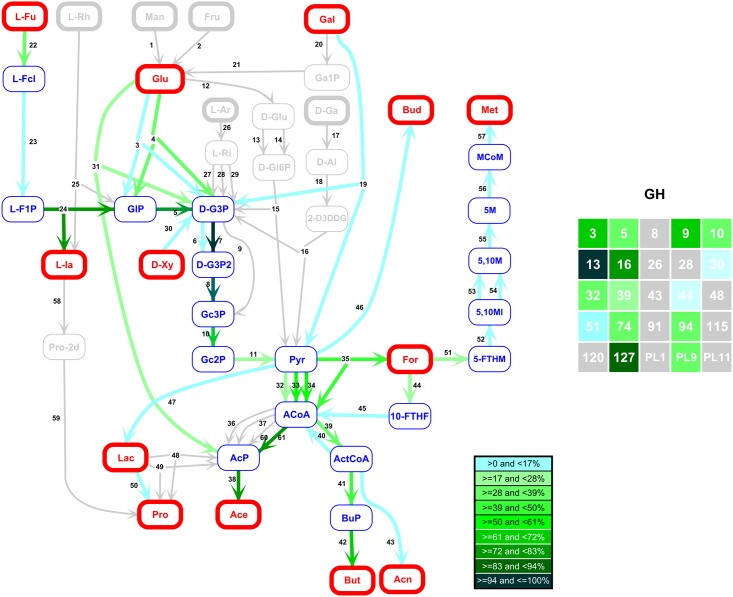
An example of CAFT. The first 61 coordinates of the functional marker frequency vector given by the first line of H*, associated to simple sugar fermentation, is represented on the reaction graph. The color scale represents percentages of the maximum coordinate among the 61 (reaction 7). The reactions form coherent pathways. The 25 coordinates associated with hydrolysis are presented in the table on the right. The numbers indicate GH families the color are scaled as percentages of the maximum coordinate among the 25 (*GH*13).

The columns of H* provide the composition of CAFTs. As an example, we detail here one of the CAFT presented in Fig 7. The 3 main genes encoding for enzyme involved in fibers hydrolysis are Glycosyl hydrolases GH13, GH16 and GH127 (Fig 7, GH table). The GH13 family is an alpha-amylase, widely represented in human intestinal microorganisms, processing resistant starch and glucose-based polysaccharides. GH16 family members are involved in the breakdown of galactose and glucose based polymers. GH127 (EC 3.2.1.185) family are arabinofuranosidases. A less abundant gene encodes for GH3 enzyme family, broadly present in micro-organisms and performing diversity of functions such as xylanase, glucosidase and arabinofuranosidase. Consistently with the above sugars released from the fiber breakdown (glucose, xylose, galactose, arabinose), the main fermentation substrates are glucose (reactions 4, 3, 31), fucose (22), D-xylose (30) and to a less extent, galactose (19). Fermentation further proceeds through the Emben-Meyerhoff-Parnas pathway to pyruvate, yielding the end products short chain fatty acids, mostly acetate, butyrate (which are the main SCFA measured in healthy human fecal samples [31]). Another major end product is lactaldehyde, resulting from the fermentation of fucose.

The hydrogenotrophic production of methane from H_2_ and CO_2_ is obtained (reactions 52 to 57). This production of methane from CO_2_ (KEGG pathway module M00567) is only restricted to hydrogenotrophic methanogens, known to be present in more than 50% of human adult population [32].

## Discussion

We presented a new NMF based approach to investigate metabolic processes in microbial ecosystems using quantitative metagenomic data. As mentioned in the introduction, NMF techniques were previously used as a “soft” clustering tool to compare samples from various ecosystems, ranging from marine environment to human body ([15, 16], [17]). However, our work focused on different aspects of NMF. Rather than clustering biological samples, we aimed at extracting biologically relevant features associated to a metabolic process, based on ecological modelling. Therefore our main interest, from the mathematical point of view, was the inference of a biologically interpretable trait matrix *H* rather than sample analysis through the weight matrix *W*.

We proposed to model the functional diversity of an habitat-specific microbial ecosystem as a mixture of contributions from subcommunities, each characterized by a profile of functional marker frequencies. These profiles were named Combined Aggregated Functional Traits in reference to [10], since they are measured at the community level from a random sample of microorganisms, regardless of their taxonomic identities. More precisely they result from both a combination of functional markers to form coherent and operative metabolic pathways, and an aggregation of individuals to form a subcommunity characterized by this trait.

Our main contribution was the design of a novel constrained NMF model for CAFT inference. Standard NMF approaches were popularised for biological data analysis mainly because usual dimension reduction techniques such as truncated Singular Value Decomposition (SVD), or the closely related Principal Componant Analysis (PCA) often lead to negative coefficients, which do not offer straightforward interpretations. However, while SVD or PCA provide a unique and well defined reduced dataset, optimal in the *ℓ*^2^-error sense, NMF is an intrinsically ill-posed problem with multiple solutions. Imposing constraints reflecting biological knowledge greatly helps to select a relevant solution. This was illustrated through the toy example in Fig 3, where both constrained and unconstrained NMF provided solutions leading to the same reconstructed data. However, only the constraints allow the solution that makes biological sense to be picked.

Our approach is in essence Bayesian, and aims at taking advantage of prior information, when available, to explore the data. We proposed a method that exploits knowledge about the genomic structure and metabolism to build relevant constraints. Genes frequencies are not randomly distributed in the metagenome and result from metabolic association encoded in microbial genomes. Thus, constraints resulting from this metabolic structuration scale up at the community level and should be accounted for in the determination of CAFT. We designed local constraints, only involving adjacent functional markers in the graph representation of the metabolic process. Hence the constraints are not strongly restrictive and still leave a considerable freedom in assembling reactions to build the CAFT matrix, without ever forcing hard reconstruction of pathways.

Our method is flexible since it allows imposing constraints only on parts of the metabolic process where biological knowledge is available and considered reliable. Moreover, it could be easily adapted to include other types of prior informations than the one we considered. For instance it should be possible, in contexts where more prior information is available, to design constraints on the weight matrix *W*, as proposed in [18] for microarray data analysis.

We considered a new approach for selecting the number of CAFTs, based on biological reproducibility instead of the numerical stability usually considered in literature. We proposed a criterion which evaluates the concordance between CAFTs computed on independent data sets, and combined it with classical procedures.

We implemented our approach on metagenes abundances from 1408 samples of the human gut microbiota, in order to determine CAFTs associated to fiber degradation in the distal gut. Fiber degradation was chosen because it is a major function of the gut microbial ecosystem. It involves the anaerobic fermentation of simple sugar which has been largely documented in the literature, allowing the design of constraints related to this step. The constraints were built by selecting 190 reference microbial genomes from prevalent species of the human gut microbiome, for which marker genes of anaerobic fermentation were annotated. This involved manual curation and relied on both state of the art knowledge of metabolism and accuracy of available annotations. We proposed an approach to account for missing information. In particular, genes were missing in pathways where they were expected to be present, which could be explained by annotation errors, or by the presence of alternative pathways undocumented in the literature. It could also come from unmodelled pathways, not related to fermentation, but sharing some common steps. Then, constraining missing genes markers would be an error. Our approach carefully took into account annotation, modelling and knowledge based errors by adjusting the constraints.

We found that our ecological model of CAFTs was consistent with the data, and that the metagenomic potential for fiber degradation by the human gut microbiota could be interpreted as a mixture of 4 CAFTs, shared by all samples in variable proportions. The CAFT detailed in the Results section is the one including the genes involved in methane production, which can be detected in 50% of a healthy population through a breath test. As a matter of fact, none of the three other CAFTs showed a high frequency of this set of genes responsible for the conversion of H_2_ and CO_2_ to methane. Two of them are prevalent among individuals, characterized by their richness in terms of GH and fermentation pathways. They differ slightly in GH repertoire and downstream fermentation genes, which may be linked to dietary habits. Finally a fourth profile, less prevalent among samples, might be more represented in diseased individuals. This data reduction highlighted biologically consistent functional patterns while providing a high quality preservation of the original data.

Provided that prior knowledge is available, the generic framework we designed can be applied to other metabolic processes in the gut or in other ecosystems. By acknowledging fundamental data structuration our approach enables the inference of meaningful functional traits.

## Materials and Methods

### Metagenomic data processing

#### Data and gene frequencies

1408 human faeces samples originating from 8 studies [3, 21–27] were exploited, covering several health status (Healthy, Crohn Disease, Ulcerative Colitis, Diabetes, Obesity) in donor groups from USA, Denmark, Spain, China and France.

The publicly released Integrated Gene Catalog (IGC) of 9.9 million genes from [21] was used to obtain counts, abundances and frequencies following the procedure described in [21] and [24].

#### Annotation

In order to obtain Glycosides Hydrolases (GH) and Pectines Lyases (PL) frequencies, we used Hmmer tool (http://hmmer.org/) and dbCan version3 [33] with default parameters. We first assessed dbCan annotation quality on 145 protein sequences for which a cazy annotation was available (http://www.cazy.org/) [34]. Similarity between annotation was evaluated by considering annotation from [34] as true and calculating accuracy. False positives mainly corresponded to Carbohydrate-binding modules, which we did not consider in this study. By ignoring them, we found an accuracy greater than 98%. As the results were good, the whole IGC was annotated, using the following rules in case of overlapping annotations, that is regions in an IGC gene sequence matching several GH or PL families: (1) if the size of the overlapping region did not exceed 10% of the smallest matched module size the overlap was ignored (2) otherwise, if the worst e-value between two overlapping annotations was 100 times smaller than the other, the corresponding annotation was rejected (3) in any other overlap situation both annotations were considered as erroneous and discarded. Regarding KO, we used the pre-existing KO annotation of the IGC, so that in the absence of any information on overlaps, the frequencies of genes with multiple KO annotations were dispatched in equal parts on each KO.

For each GH, PL and KO, the frequencies of all genes with the corresponding annotation were summed to obtain a global frequency.

#### Markers of fiber degradation in the human gut microbiota

We selected 25 GH and PL relevant to the human gut microbiota, involved in the degradation of dietary fiber such as cellulose, hemicellulose, starch and pectin [35]. The pathways involved in the anaerobic degradation and fermentation of sugars resulting from the hydrolysis of fiber are well characterized. They were identified using bibliographic resources [36] and Metacyc database [37] guided by expertise. Pathways exclusively associated to microorganisms unlikely present in the gut (e.g. soil microorganisms or extremophils) were excluded. A list of putative reactions and associated KO was compiled from the selected pathways using KEGG database, filtered according the following rules: (1) when a KO was not found in the IGC annotation it was ignored, (2) when a reaction could not be linked to a KO, it was ignored, (3) when the enzyme from a KO could catalyze more than one reaction, preventing from accurately linking the KO frequency to a unique target reaction, this KO was discarded as well as any other KO that could catalyze this reaction. Since rule (3) is stringent, a few KO able to catalyze several reactions were not discarded, but only when these additional reactions (and the associated pathway) were highly unlikely present in the gut microbiota (see S1 Table).

Some reactions were associated with multiple KO corresponding to either different enzymes catalyzing the same chemical reaction or parts of the same enzyme. In the first situation, each KO was considered as a functional marker, in the second situation the corresponding KO were merged into a unique marker whose frequency was set to the mean of the KO frequencies.

A final list of 86 functional markers characterizing the fiber degradation process in the human gut microbiome was obtained, comprising 25 GH and PL and 61 KO or KO aggregations (see S1 Table). The data matrix *A* was built from those 86 markers frequencies for the 1408 samples (see S1 tsv File).

### Building the linear constraint from pathway prior knowledge

#### General principles for constraint building

Considering a microorganism *x* in the ecosystem, we denote *n*_*xj*_ the number of functional markers *j* in its genome. Moreover, for each intracellular metabolite *m*, we denote by *P*_*m*_ the set of indexes of functional makers involved in reactions producing *m*, and *C*_*m*_ the set of indexes of functional makers involved in reactions consuming *m*. Then $N_{x,m,P}\;=\;\sum_{j\in P_{m}}$ *n*_*xj*_ is the number of markers associated to the production of metabolite *m* in the genome of *x*, and $N_{x,m,C}\;=\;\sum_{j\prime \in C_{m}}$ *n*_*xj*′_ denotes the number of markers associated to the consumption of *m*.

If *N*_*x*,*m*,*C*_ is positive, we can define the ratio *δ*_1,*x*,*m*_ = *N*_*x*,*m*,*P*_/*N*_*x*,*m*,*C*_, so that the following equality holds

$$N_{x,m,P}=\delta_{1,x,m}N_{x,m,C}$$ (11)

and in the same way, if *N*_*x*,*m*,*P*_ is positive, we can define *δ*_2,*x*,*m*_ = *N*_*x*,*m*,*C*_/*N*_*x*,*m*,*P*_, and write

$$N_{x,m,C}=\delta_{2,x,m}N_{x,m,P}.$$ (12)

If *N*_*x*,*m*,*C*_ and *N*_*x*,*m*,*P*_ are both zero, we arbitrarily set *δ*_1,*x*,*m*_ = *δ*_2,*x*,*m*_ = 0 and both Eqs (11) and (12) hold.

By definition, a CAFT *l* characterizes a gene pattern associated in each sample *i* to a subcommunity *C*_*il*_ in which the total abundance of any functional marker *j* is proportional to *h*_*lj*_, namely

$$\sum_{x\in C_{il}}n_{xj}=w_{il}h_{lj}.$$ (13)

Therefore, for all *m*, $\sum_{x\in C_{i,l}}\;N_{x,m,P}\;=\;w_{il}\;\sum_{j\in P_{m}}\;h_{lj}$ and $\sum_{x\in E_{i,l}}\;N_{x,m,C}\;=\;w_{il}\;\sum_{j\in C_{m}}\;h_{lj}$.

Consider now a metabolite *m* for which Eq (11) holds for all microorganisms *x* in the ecosystem, and define *δ*_1,*m*_ = max_*x*_ *δ*_1,*x*,*m*_. Therefore *N*_*x*,*m*,*P*_ ≤ *δ*_1,*m*_ *N*_*x*,*m*,*C*_ and for each CAFT *l*, *h*_*l*_ satisfies

$$\sum_{j\in P_{m}}h_{lj}\leq \delta_{1,m}\sum_{j^{\prime }\in C_{m}}h_{lj^{\prime }}$$ (14)

In the same way, if we consider a metabolite *m* for which Eq (12) holds for all microorganisms *x* in the ecosystem, each CAFT *l* satisfies

$$\sum_{j^{\prime }\in C_{m}}h_{lj^{\prime }}\leq \delta_{2,m}\sum_{j\in P_{m}}h_{lj}$$ (15)

where *δ*_2,*m*_ = max_*x*_ *δ*_2,*x*,*m*_.

In practice, for complex microbial ecosystems, it is not possible to compute the values of *δ*_1,*m*_ and *δ*_2,*m*_, since no exhaustive knowledge about the microorganisms that are present and their genomes is available. However it is possible to propose sensible values that can be used in Eqs (14) and (15), as shown below in the case of fiber degradation by the gut ecosystem.

For a convenient implementation of the constraints, we built a graph representing the structure of the reactions under consideration, in which each vertex is a metabolite and each edge is directed and corresponds to a functional marker associated to a reaction. We built a *m*_*int*_ × *r* matrix *Q* derived from the incidence matrix of the graph. For each intracellular metabolite *m* and each functional marker *j*, *q*_*mj*_ = 1 (resp. *q*_*mj*_ = −1) if the reaction associated with functional marker *j* produces (resp. consumes) metabolite *m*, and *q*_*mj*_ = 0 otherwise. Thus, columns corresponding to functional markers not included in the graph are equal to zero. The matrix *Q* was split as *Q* = *Q*^+^ − *Q*^−^, where *Q*^+^ contains the positive terms in *Q* and *Q*^−^ the absolute value of the negative ones. Note that

$$\sum_{j\in P_{m}}h_{lj}=q_{m}^{+}h_{l}\;\text{and}\;\sum_{j\in C_{m}}h_{lj}=q_{m}^{-}h_{l}$$

where $q_{m}^{+}$ and $q_{m}^{-}$ respectively denote the *m*^*th*^ line of *Q*^+^ and *Q*^−^.

We define *M*_1_ and *M*_2_ as the two sets of intracellular metabolites for which constraints (14) and (15) respectively apply. The rows corresponding to sets *M*_1_ and *M*_2_ were extracted from *Q*^+^ and *Q*^−^ and four sub-matrices $Q_{i}^{+}$ and $Q_{i}^{-}$ for *i* = 1, 2 were built. Then constraints (14) and (15) were formulated as

$$F_{\Delta }H^{T}=\left(\begin{array}{c} Q_{1}^{+}-\Delta_{1}Q_{1}^{-}\\ Q_{2}^{-}-\Delta_{2}Q_{2}^{+} \end{array}\right)H^{T}\leq 0$$ (16)

where Δ_1_ and Δ_2_ were diagonal matrices with diagonal entries *δ*_1,*m*_ and *δ*_2,*m*_ for *m* in *M*_1_ and *M*_2_ respectively.

#### Constraint building for fiber degradation by the gut microbiota

We considered two types of functional markers, GH/PL for fiber degradation into simple sugars and KO for anaerobic fermentation of simple sugars. While precise information is available for anaerobic fermentation, the GH/PL families are not substrate specific, thus although they are essential in the fiber degradation process, they could not be included in a reaction graph. Based on the KO only, we built an oriented graph from simple sugars to SCFA. Edge orientation represents the biological catabolic pathway from simple sugars to SCFA and methane. During the KO selection process, several KO did not pass the specificity criterion and were discarded, preventing fully continuous pathways from sugars to short chained fatty acids and simplifications were introduced in order to build a connected graph. We finally obtained a graph with 43 nodes corresponding to metabolites (see Table 1 for their complete and abbreviated names) and *r*_1_ = 61 edges corresponding to the selected KO (see Table 2). The metabolites were divided into *m*_*int*_ = 25 intracellular and *m*_*ext*_ = 18 extracellular ones. A summary of simplification, selected metabolites and KO is available in S1 Table. The list of selected GH/PL is provided in Table 3.

Approximate values for the entries of Δ_1_ and Δ_2_ were computed by screening 190 core genomes (see S2 tsv File) chosen from [22] and [38], which were annotated using blast on Kegg Orthologies with a bitscore threshold of 60. The number of copies of the *r* selected markers for each genome were gathered in a 190 × *r* matrix denoted *G*. We denote *n*_*x*_ the *x*^*th*^ row of *G* containing the number of copies of the functional markers in genome *x*, so that according to the notations defined above, $N_{x,m,C}=q_{m}^{-}n_{x}$ and $N_{x,m,P}=q_{m}^{+}n_{x}$.

In order to build an approximated set ${\tilde{M}}_{1}$, we first relaxed the theoretical definition of *M*_1_ given in the Results section to make it more realistic. Indeed, for a given intracellular metabolite *m*, if there was only a small fraction of genomes *x* such that *N*_*x*,*m*,*C*_ = 0, we assumed that they could be neglected since we are looking for bounds on markers in aggregated populations.

Then we adopted the following empirical rules:

*Rule 1*: If *N*_*x*,*m*,*C*_ > 0 for more than 95% of the 190 core genomes, meaning that *N*_*x*,*m*,*C*_ = 0 for 9 genomes or less, then *m* is included in ${\tilde{M}}_{1}$.
*Rule 2*: If *N*_*x*,*m*,*C*_ > 0 for less than 85% of the 190 core genomes, meaning that *N*_*x*,*m*,*C*_ = 0 for 28 genomes or more, then *m* is not included in ${\tilde{M}}_{1}$.
*Rule 3*: If the proportion of genomes for which *N*_*x*,*m*,*C*_ > 0 is between 85% and 90%, then we check if *m* is specifically involved in anaerobic sugar fermentation, or if it could be involved in other metabolic processes. If no specificity is found, then *m* is not included in ${\tilde{M}}_{1}$, otherwise it is stored in a set *S*_1_.
  The same rules were applied to build ${\tilde{M}}_{2}$ and *S*_2_, upon replacement of *N*_*x*,*m*,*C*_ by *N*_*x*,*m*,*P*_.

An additional selection step was performed according to:

*Rule 4*: If *m* ∈ *S*_1_∪*S*_2_, meaning that either the proportion of genomes for which *N*_*x*,*m*,*C*_ > 0 or the proportion of genomes for which *N*_*x*,*m*,*P*_ > 0 is between 85% and 90%, then if *m* ∈ *S*_1_ and *m* ∉ *S*_2_ it is included in ${\tilde{M}}_{1}$, if *m* ∈ *S*_2_ and *m* ∉ *S*_1_ it is included in ${\tilde{M}}_{2}$ and if *m* ∈ *S*_1_ ∩ *S*_2_ it is neither included in ${\tilde{M}}_{1}$ nor in ${\tilde{M}}_{2}$.

After applying these rules, based on biological expertise, it was additionally considered that the metabolism of the Propane 1,2-diol was not well known and it was neither included in ${\tilde{M}}_{1}$ nor in ${\tilde{M}}_{2}$

Table 5 summarizes the affectation process of all the intracellular metabolites for which either *N*_*x*,*m*,*C*_ = 0 or *N*_*x*,*m*,*P*_ = 0 for 10 genomes or more. All the other metabolites were included both in ${\tilde{M}}_{1}$ and ${\tilde{M}}_{2}$.

**Table 5:** Affectation in ${\tilde{M}}_{1}$ and ${\tilde{M}}_{2}$ of metabolites for which Rule 1 is false.

Metabolite	Nb genomes s.t. hypothesis is false	Hypothesis	Membership	Rule
Acetoacetyl-CoA	34	*N*_*x*,*m*,*C*_ > 0	$\notin {\tilde{M}}_{1}$	Rule 2
ButanoylP	28	*N*_*x*,*m*,*C*_ > 0	$\in {\tilde{M}}_{1}$	Rule 2
D-Altronate	10	*N*_*x*,*m*,*C*_ > 0	$\in {\tilde{M}}_{1}$	Rule 4
D-Gluconate6P	12	*N*_*x*,*m*,*C*_ > 0	$\notin {\tilde{M}}_{1}$	Rule 4
D-gluconolactone	12	*N*_*x*,*m*,*C*_ > 0	$\notin {\tilde{M}}_{1}$	Rule 4
Galactose1P	68	*N*_*x*,*m*,*C*_ > 0	$\notin {\tilde{M}}_{1}$	Rule 2
L-Fuculose1P	14	*N*_*x*,*m*,*C*_ > 0	$\notin {\tilde{M}}_{1}$	Rule 4
2-D3DDG	89	*N*_*x*,*m*,*P*_ > 0	$\notin {\tilde{M}}_{2}$	Rule 2
D-Gluconate6P	38	*N*_*x*,*m*,*P*_ > 0	$\notin {\tilde{M}}_{2}$	Rule 4
D-gluconolactone	24	*N*_*x*,*m*,*P*_ > 0	$\notin {\tilde{M}}_{2}$	Rule 4
L-Fuculose	17	*N*_*x*,*m*,*P*_ > 0	$\in {\tilde{M}}_{2}$	Rule 4
L-Fuculose1P	26	*N*_*x*,*m*,*P*_ > 0	$\notin {\tilde{M}}_{2}$	Rule 4
L-Ribulose	27	*N*_*x*,*m*,*P*_ > 0	$\notin {\tilde{M}}_{2}$	Rule 3
Propanal			$\notin {\tilde{M}}_{2}\cup {\tilde{M}}_{2}$	expertise

Finally, from the initial 25 intracellular metabolites, only 21 were used to generate contraints on the CAFTs. 17 metabolites were included both in ${\tilde{M}}_{1}$ and ${\tilde{M}}_{2}$, 2 in ${\tilde{M}}_{1}$ and not in ${\tilde{M}}_{2}$ and 2 in ${\tilde{M}}_{2}$ and not in ${\tilde{M}}_{1}$, resulting in a total of 38 constraints.

For each *m* in ${\tilde{M}}_{1}$ (resp ${\tilde{M}}_{2}$), we denoted *E*_*m*_ (resp *F*_*m*_) the set of genomes *x* such that *N*_*x*,*m*,*C*_ > 0 (resp *N*_*x*,*m*,*P*_ > 0). Then we defined

$$\delta_{1,m}^{0}=1.5\max_{x\in E_{m}}\left(\frac{N_{x,m,P}}{N_{x,m,C}}\right)$$

and for each m in *M*_2_:

$$\delta_{2,m}^{0}=1.5\max_{x\in F_{m}}\left(\frac{N_{x,m,C}}{N_{x,m,P}}\right)$$

We further chose to define 5 by 5 integer thresholds

$$\begin{array}{c} \delta_{1,m}=5\left\lceil \frac{\delta_{1,m}^{0}}{5}\right\rceil \\ \delta_{2,m}=5\left\lceil \frac{\delta_{2,m}^{0}}{5}\right\rceil \end{array}$$

This rounding step is somewhat redundant with the multiplicative factor, in complex situations with many functional markers as here we propose to use it to cluster the constraints by similar order of magnitude, but it may be omitted. Table 4 displays the values found for each *m*, when relevant.

Finally, Eq (16) represents a set of 38 constraints with entries of Δ_1_ and Δ_2_ ranging from 5 to 30. The resulting matrix *F*_Δ_ is given in S3 tsv File.

### Inference

#### Constrained NMF problem formulation and inference procedure

To take into account discrepancies among reactions, the (*n* × *r*) matrix *A* is scaled per reaction according to the formula

$${\tilde{a}}_{ij}=\frac{a_{ij}}{\sqrt{\sum_{p=1}^{n}\left(a_{pj}^{2}\right)}}$$ (17)

were the scaled abundance matrix is denoted $\tilde{A}$. Defining the scaling matrix *D* as the diagonal matrix whose *j*^*th*^ diagonal entry is $\sqrt{\sum_{p=1}^{n}\left(a_{pj}^{2}\right)}$, it follows that $\tilde{A}=AD^{-1}$, and the trait matrix has to be scaled accordingly as $\tilde{H}=HD^{-1}$. The approximation problem is therefore formulated and solved in terms of matrix *W* and $\tilde{H}$, after which *H* is recovered as $H=\tilde{H}D$.

The approximation error is defined using the Frobenius norm as given in Eq (9). Following [12] we use a regularizing term aimed at encouraging sparsity over the columns of $\tilde{H}$, formulated as $\alpha (\|W{\|}_{F}^{2}+\|1^{T}\tilde{H}{\|}_{2}^{2})$, where 1 is defined as

$$1=\underset{\text{size}\;\text{k}}{\underset{︸}{{\left(1,\;1,\ldots ,1\right)}^{T}}}$$ (18)

More details about this regularization term is given in S1 Text.

The matrix ${\tilde{F}}_{\Delta }=F_{\Delta }D^{T}$ such that $F_{\Delta }H^{T}={\tilde{F}}_{\Delta }{\tilde{H}}^{T}$ is defined, in order to translate the constraints in terms of $\tilde{H}$. Finally, for a given value of (*k*, *α*) the NMF problem to be solved is formulated as:

$$\begin{array}{c} \left(W^{⋆},{\tilde{H}}^{⋆}\right)=\underset{W,\tilde{H}}{\;\text{argmin}}\;\|\tilde{A}-W\tilde{H}{\|}_{F}^{2}+{\alpha (\|W\|}_{F}^{2}+\|\tilde{H}{\|}_{1,2}^{2})\\ s.t.\;W\geq 0,\;\tilde{H}\geq 0,\;{\tilde{F}}_{\Delta }{\tilde{H}}^{T}\leq 0 \end{array}$$ (19)

where $\tilde{H}$ is a (*k* × *r*) matrix and *W* is a (*n* × *r*) matrix. The NMF decomposition of *A* is (W*, H*) with $H^{*}={\tilde{H}}^{*}D$.

#### Numerical implementation

Algorithm 1 details the inference process for the determination of *W*^⋆^ and ${\tilde{H}}^{⋆}$. The minimization step (20) is carried out using a block-coordinate descent algorithm detailed in Algorithm 2, in which *W* and $\tilde{H}$ are alternatively updated.

**Algorithm 1** NMF resolution

$\tilde{A}\leftarrow AD^{-1}$

${\tilde{F}}_{\Delta }\leftarrow F_{\Delta }D^{T}$

**for** Iter = 1 to 5 **do**

$\left(W_{0,\text{Iter}},{\tilde{H}}_{0,\text{Iter}})∼u(0,1\right)$

$W_{\text{ini},\text{Iter}}\leftarrow W_{0,\text{Iter}}\sqrt{\frac{\|\tilde{A}{\|}_{F}}{\|W_{0,\text{Iter}}{\tilde{H}}_{0,\text{Iter}}{\|}_{F}}}$

${\tilde{H}}_{\text{ini},\text{Iter}}\leftarrow {\tilde{H}}_{0,\text{Iter}}\sqrt{\frac{\|\tilde{A}{\|}_{F}}{\|W_{0,\text{Iter}}{\tilde{H}}_{0,\text{Iter}}{\|}_{F}}}$

$$\begin{array}{c} \left(W_{\text{Iter}}^{⋆},{\tilde{H}}_{\text{Iter}}^{⋆}\right)=\underset{W,\tilde{H}}{\text{argmin}}\;\|\tilde{A}-W\tilde{H}{\|}_{F}^{2}+{\alpha (\|W\|}_{F}^{2}+\|\tilde{H}{\|}_{1,2}^{2})\\ s.t.\;W\geq 0,\;\tilde{H}\geq 0,\;{\tilde{F}}_{\Delta }{\tilde{H}}^{T}\leq 0 \end{array}$$ (20)

**end for**

$(W^{⋆},{\tilde{H}}^{⋆})\leftarrow \underset{\text{Iter}\in [1,5]}{\text{argmin}}\|\tilde{A}-W_{\text{Iter}}^{⋆}{\tilde{H}}_{\text{Iter}}^{⋆}{\|}_{F}^{2}$

$H^{⋆}\leftarrow {\tilde{H}}^{⋆}D$

**Algorithm 2** Block-Coordinate-Descent for NMF

**while** Convergence ≠ True **do**

$${\tilde{H}}^{\left(t+1\right)}\leftarrow \underset{H\geq 0,F_{\Delta }H^{T}\leq 0}{\text{argmin}}\;\|\tilde{A}-W^{t}H{\|}_{F}^{2}+\alpha \|H{\|}_{1,2}^{2}$$ (21)

$$W^{\left(t+1\right)}\leftarrow \underset{W\geq 0}{\text{argmin}}\;\|\tilde{A}-W{\tilde{H}}^{t+1}{\|}_{F}^{2}+\alpha \|W{\|}_{F}^{2}$$ (22)

**end while**

Algorithm 2 was implemented in C language. The minimization step (22) is performed using Nesterov’s first order method (see [39]). Step (21) is solved using a standard Augmented Lagrangian Method [40], in which Uzawa iterations are combined with Nesterov’s first order method (see S2 Text for pseudo-code). Finally, upon convergence, the NMF approximation of $\tilde{A}$ is (*W*^⋆^, *H*^⋆^) defined in Algorithm 1.

#### Criteria for parameter selection

According to the algorithm presented in Numerical Implementation section, each NMF decomposition (on all or part of the data set) involved in the following criteria was computed using 5 random initializations and selecting the decomposition which leads to the smallest reconstruction error. The range of values of *k* on which the criteria are computed is determined by inspecting the SVD decomposition of $\tilde{A}$, as well as the size of the data over parameter ratio *nr*/(k*(*n*+*r*)).

*Bi-cross-validation.* The bi-cross-validation procedure developed in [20] is an adaptation of the classic cross-validation procedure to the NMF framework, in which both samples (rows of *A*) and functional markers (columns of *A*) are split into training and validation sets. Bi-cross-validation error evaluates the prediction ability of the NMF decomposition. Nevertheless, it can not be computed for constrained NMF inference since the column split breaks up the structure of the metabolic relationships on which the constraints are based. Therefore, we consider this criterion as an indication of the performances of the NMF decomposition computed without constraints.

*Reconstruction error.* The reconstruction error ‖*A* − W* H*‖_*F*_/‖*A*‖_*F*_ indicates the proportion of information in *A* recovered in the NMF approximation W* H*. For a fixed *α*, this quantity automatically decreases as the number of profiles *k* increases, and the decreasing rate when a profile is added indicates the significance of this profile. Thus, a slope discontinuity on the graph of the reconstruction error as a function of *k* indicates that additional profiles are less significant. Similarly, for a given *k* the reconstruction error increases as *α* increases; we considered the optimal value to be the largest *α* before the reconstruction error significantly increases.

*Concordance of CAFTs from independent data sets.* Let *M* be a matrix of dimension (*n*_1_, *r*_1_), the similarity matrix *S*^*M*^ of *M* is the squared matrix with *r*_1_ columns whose (*j*, *j*′) entry is the cosine of the angle between the column vectors *j* and *j*′ of *M*:

$$s_{jj^{\prime }}^{M}=\frac{\sum_{l=1}^{n_{1}}m_{lj}m_{lj^{\prime }}}{{\left(\sum_{l=1}^{n_{1}}m_{lj}^{2}\right)}^{1/2}{\left(\sum_{l=1}^{n_{1}}m_{lj^{\prime }}^{2}\right)}^{1/2}}.$$

For repetitions *a* = 1, …, 40, the set of rows of $\tilde{A}$ is randomly split in two independent sets of equal length, the corresponding submatrices ${\tilde{A}}_{1}^{\left(a\right)}$ and ${\tilde{A}}_{2}^{\left(a\right)}$ are extracted and their NMF decompositions $\left(W_{1}^{\left(a\right)},{\tilde{H}}_{1}^{\left(a\right)}\right)$ and $\left(W_{2}^{\left(a\right)},{\tilde{H}}_{2}^{\left(a\right)}\right)$ are inferred using the constrained NMF algorithm. Then, the concordance index is defined as

$$1-\frac{1}{40\times \sqrt{r(r-1)}}\sum_{a=1}^{40}{\left\|S_{1}^{\left(a\right)}-S_{2}^{\left(a\right)}\right\|}_{F}$$

with $S_{1}^{\left(a\right)}$ and $S_{2}^{\left(a\right)}$ the similarity matrices of ${\tilde{H}}_{1}^{\left(a\right)}$ and ${\tilde{H}}_{2}^{\left(a\right)}$.

*Selection of optimal parameters for fiber digestion analysis.* The optimal value of *α* was selected by computing the three criteria for (*α*, *k*) in (10^−3^, 10^−2^, 10^−1.5^, 10^−1^) × (2, 4, 6, 8, 11, 13). Then, the optimal number of CAFTs *k* was selected by computing the three criteria for the optimal value of *α* and for *k* in the set of integers from 2 to 15. The same 10-fold split of rows and columns in the bi-cross-validation procedure, and the same row splits for concordance of *H*, were used for all values of *k* and *α*.

#### Sensitivity to the constraints

We performed additional inferences with *k* = 4 in order to assess the sensitivity of the resulting matrices to the thresholds used to define the constraints. We tested three pairs of thresholds in Rules 1, 2 and 3 used to build the constraints, namely [0%, 10%], [5%, 15%] and [10%, 30%]. These parameters influence the number or nodes (metabolites) included in the sets ${\tilde{M}}_{1}$ and ${\tilde{M}}_{2}$: the larger the threshold values, the more metabolites are included in these sets, and therefore the more constraints are imposed to the functional markers frequencies. For each of these pairs, we tested three options for the computation of the parameters *δ*_1,*m*_ and *δ*_2,*m*_ associated to each constraint. The first option was to compute them as the value obtained on the set of 190 genomes, with a multiplicative factor of 1 and no rounding. The second option was the one presented in the paper: the values obtained from the genomes are multiplied by 1.5 and rounded to the nearest upper multiple of 5. The third rule was the same as the second one with a multiplicative factor of 2. So the resulting constraints rank from the more stringent to the less stringent. The sensitivity was assessed by measuring the concordance index between the inferred CAFT matrices and the CAFT matrix obtained with an unconstrained NMF. We observed that the results were mainly influenced by the thresholds, and that the sensitivity to the computation rule tended to be weaker. For the pair [0%, 10%] the deviation from the unconstrained case was very weak (less than 1% on average), for the pair [5%, 15%] the deviation was small (less than 5% on average) and it was higher for the pair [10%, 30%] (12% on average). Therefore the choice [5%, 15%] seems to be sensible on this example.

### Preservation of the geometric struture

The columns of matrices *A* and *W* were *ℓ*^1^-normalized; the normalized matrices correspond to the repartition of the functional markers (resp. the CAFTs) abundancies among the samples. The distance matrix *d*^*A*^ (resp. *d*^*W*^) of *A* (resp. *W*) defined as the 1408 × 1408 matrix whose element (*i*, *j*) is the euclidean distance between rows *i* and *j* of *A* (resp. *W*) was computed. For each biological sample *i*, the Pearson correlation between the distance of *i* to the other samples in *A* and in *W* was evaluated:

$$C_{i}=\text{cor}\left({\left(d_{i,j}^{A}\right)}_{j\neq i},{\left(d_{i,j}^{W}\right)}_{j\neq i}\right)$$

and the histogram of (*C*_*i*_)_*i* = 1, …, 1408_ was displayed.

## Supporting Information

S1 Text Regularization term. This file details the mathematical motivation for using a unique regularization parameter.
(PDF)

S2 Text Pseudo-code of the minimization algorithm. This file provides details about optimization steps (21) and (22).
(PDF)

S3 Text Tuning of the regularization parameter *α*. This file details the selection of the regularization parameter *α* based on the three criteria.
(PDF)

S1 tsv File Functional markers abundances. This file contains the abundance matrix of the 86 functional markers of fiber degradation in 1408 samples.
(TSV)

S2 tsv File List of 190 core genomes used for constraint building. (TSV)

S3 tsv File Constraint matrix. This file contains the constraint matrix *F*_Δ_, storing the coefficients of the 38 constraints on the 86 entries of the CAFTs.
(TSV)

S1 Table Description of fiber hydrolysis. This file details the hypotheses underlying the selection of functional markers and metabolites representing simple sugar anaerobic fermentation in the human gut (sheet Reactions and KOs) and provides the list of the GH and PL selected in the model (sheet GH and PL).
(XLSX)
