# Supplementary material for: Inferring Aggregated Functional Traits from Metagenomic Data Using Constrained Non-negative Matrix Factorization: Application to Fiber Degradation in the Human Gut Microbiota
Source: PLoS Comput Biol. 2016 Dec 16;12(12):e1005252. doi: 10.1371/journal.pcbi.1005252 (PMC5161307; doi:10.1371/journal.pcbi.1005252)
Supplement: S1 Text — This file details the mathematical motivation for using a unique regularization parameter. (PDF) [file pcbi.1005252.s001.pdf]

Designing the regularization term in the NMF problem

From now on, we consider the normalized matrices and drop the tilde notation for A and H. For any positive integers n and p, we denote $\mathcal{M}_{n,p}(\mathbb{R}^+)$ the set of matrices with n lines, p columns and non-negative entries. Let us set

$$C = \{(W, H) \in \mathcal{M}_{n,k}(\mathbb{R}^+) \times \mathcal{M}_{k,r}(\mathbb{R}^+)\ ,\ F_\Delta H^t \leq 0\}.$$

As soon as C is not reduced to $(0, 0)$, there are an infinity of matrices $(W, H) \in C$ with the same product WH. For example, let $(W, H) \in C$, dividing W and multiplying H by the same positive constant produces matrices in C and preserve the product WH. The NMF is therefore an ill-posed problem. Adding regularization terms on W and H in the NMF inference process should help to restrain the set of solutions of the minimization problem, to encourage some specific features such as sparsity and to decrease significantly the computation time.

We first impose a regularization term on H , defined as

$$\alpha\|H\|_{1,2}^2 = \alpha\|\mathbf{1}^t H\|_2^2 = \alpha\left(\sum_{j=1}^{r}\left(\sum_{l=1}^{k} h_{l,j}\right)^2\right),$$

where α is a positive parameter and $\mathbf{1} \in \mathbb{R}^{k\times 1}$ a vector of 1. For a fixed W, this regularization acts independently on each column of H. Indeed, solving

$$\min_{\boldsymbol{H}\geq 0,\, F_\Delta \boldsymbol{H}^t \geq 0} \|A - W\boldsymbol{H}\|_F^2 + \alpha\|\mathbf{1}^t\boldsymbol{H}\|_2^2$$

is equivalent to the minimization of the r following independent sub-problems

$$\forall j \in [\![1, r]\!],\ \min_{\boldsymbol{H}\geq 0, F_\delta \mathbf{H^t}\geq \mathbf{0}} \|a_{:,j} - W\mathbf{h_{:,j}}\|_2^2 + \alpha\|\mathbf{h_{:,j}}\|_1^2.$$

Here we use the convenient $(:, j)$ notation to refer to the jth column of matrices A and

H. This type of regularization specifically promotes sparsity across the columns of H (functional markers), via the squared ℓ^1-norm of each column. It therefore encourages functionally distinct CAFTs.

The regularization term on W is quadratic,

$$\beta \|W\|_F^2,$$

with β a positive parameter.

The regularized criterion to be minimized in the constraint domain C is therefore expressed as

$$T(W, H, \alpha, \beta) = \|A - WH\|_F^2 + \alpha \|\mathbf{1}^t H\|_2^2 + \beta \|W\|_F^2.$$

We now show that a single regularization parameter α is needed (Proposition 2) and that the role of the second one is to specify the ratio between the norms of matrices W and H (Proposition 3). We start with the following technical proposition.

Proposition 1 *For all $\alpha, \beta > 0$, let $\boldsymbol{a} = (\beta/\alpha)^{1/4}$, then*

$$T(W, H, \alpha, \beta) = T(\boldsymbol{a}W, H/\boldsymbol{a}, \sqrt{\alpha\beta}, \sqrt{\alpha\beta}).$$

Proof Let $\alpha, \beta > 0$, by definition of $\boldsymbol{a}$, $\sqrt{\alpha\beta}\boldsymbol{a}^2 = \beta$ and $\sqrt{\alpha\beta}/\boldsymbol{a}^2 = \alpha$. Therefore

$$\begin{aligned}
& T(\boldsymbol{a}W, H/\boldsymbol{a}, \sqrt{\alpha\beta}, \sqrt{\alpha\beta}) \\
= \; & \|A - \boldsymbol{a}WH/\boldsymbol{a}\|_F^2 + \sqrt{\alpha\beta}/\boldsymbol{a}^2 \|\mathbf{1}^t H\|_2^2 + \sqrt{\alpha\beta}\boldsymbol{a}^2 \|W\|_F^2 \\
= \; & \|A - WH\|_F^2 + \alpha \|\mathbf{1}^t H\|_2^2 + \beta \|W\|_F^2 \\
= \; & T(W, H, \alpha, \beta).
\end{aligned}$$

The following result is an immediate consequence of Proposition 1.

Proposition 2 *For all $\alpha, \beta > 0$, let $\boldsymbol{a} = (\beta/\alpha)^{1/4}$. Then if $(W^\star, H^\star)$ is a local (resp. global) solution of*

$$\min_{(W,H) \in C} T(W, H, \alpha, \beta) \tag{1}$$

then $(\boldsymbol{a}W^\star, H^\star/\boldsymbol{a})$ *is a local (resp. global) solution of*

$$\min_{(W,H)\in C} T(W, H, \sqrt{\alpha\beta}, \sqrt{\alpha\beta}). \tag{2}$$

Proof Let $(W^\star, H^\star)$ be a local solution of (1), then by definition, there exists $\rho \in \mathbb{R}^{+*}$ such that for all $(W, H) \in \mathcal{B}((W^\star, H^\star), \rho) \cap C$, where $\mathcal{B}((W^\star, H^\star), \rho)$ is the open ball of radius ρ centred on $(W^\star, H^\star)$,

$$T(W, H, \alpha, \beta) \geq T(W^\star, H^\star, \alpha, \beta).$$

By Proposition 1, for all $(W, H) \in \mathcal{B}((W^\star, H^\star), \rho) \cap C$,

$$T(\boldsymbol{a}W, H/\boldsymbol{a}, \sqrt{\alpha\beta}, \sqrt{\alpha\beta}) \geq T(\boldsymbol{a}W^\star, H^\star/\boldsymbol{a}, \sqrt{\alpha\beta}, \sqrt{\alpha\beta}). \tag{3}$$

Besides, the transformation $(W, H) \mapsto (\boldsymbol{a}W, H/\boldsymbol{a})$ is a bijection from C to C. Moreover, let $\rho' = \min(\boldsymbol{a}, 1/\boldsymbol{a})\rho$, then the inverse transformation $(W', H') \mapsto (W'/\boldsymbol{a}, \boldsymbol{a}H')$ maps the set $\mathcal{B}((\boldsymbol{a}W^\star, H^\star/\boldsymbol{a}), \rho') \cap C$ to a subset of $\mathcal{B}((W^\star, H^\star), \rho) \cap C$. According to (3), all $(W', H') \in \mathcal{B}((W^\star, H^\star), \rho') \cap C$ satisfies

$$T(W', H', \sqrt{\alpha\beta}, \sqrt{\alpha\beta}) \geq T(\boldsymbol{a}W^\star, H^\star/\boldsymbol{a}, \sqrt{\alpha\beta}, \sqrt{\alpha\beta}). \tag{4}$$

Therefore $(\boldsymbol{a}W^\star, H^\star/\boldsymbol{a})$ is a local solution of (2). The proof for a global solution is identical upon replacement of $\mathcal{B}((W^\star, H^\star), \rho)$ and $\mathcal{B}((\boldsymbol{a}W^\star, H^\star/\boldsymbol{a}), \rho')$ by C.

Moreover, the following proposition holds.

Proposition 3 *Let* $(W^\star, H^\star)$ *be a local minimum of* $T(W, H, \alpha, \beta)$ *in* C*, then*

$$\alpha \|\boldsymbol{1}^t H^\star\|_2^2 = \beta \|W^\star\|_F^2$$

Proof Let $\rho \in \mathbb{R}^{+*}$ be such that for all (W, H) in $\mathcal{B}((W^\star, H^\star), \rho) \cap C$,

$$T(W, H, \alpha, \beta) \geq T(W^\star, H^\star, \alpha, \beta).$$

The proof is by contradiction. Suppose that there exists a constant $\kappa \neq 1$ such that

$$\kappa^2 \alpha \|\mathbf{1}^t H^\star\|_2^2 = \beta \|W^\star\|_F^2$$

For any $\lambda > 0$ we define $W_\lambda = \lambda W^\star$ and $H_\lambda = H^\star/\lambda$. Then $(W_\lambda, H_\lambda) \in C$ and

$$\begin{aligned} & T(W_\lambda, H_\lambda, \alpha, \beta) \\ = \; & \|A - W^\star H^\star\|_F^2 + \frac{\alpha}{\lambda^2} \|\mathbf{1}^t H^\star\|_2^2 + \beta \lambda^2 \|W^\star\|_F^2 \\ = \; & \|A - W^\star H^\star\|_F^2 + \left(1/\lambda^2 + \lambda^2 \kappa^2\right) \alpha \|\mathbf{1}^t H^\star\|_2^2. \end{aligned}$$

Consider the function $f(\lambda) = (\lambda^2 \kappa^2 + 1/\lambda^2)$ and its derivative $f'(\lambda) = (2\lambda(\kappa^2 - 1/\lambda^4))$. Obviously if $\kappa \neq 1$ then $f'(1) \neq 0$, therefore there exists $\lambda_0 > 0$ such that $\lambda_0 \neq 1$ and

$$(W_{\lambda_0}, H_{\lambda_0}) \in \mathcal{B}((W^\star, H^\star), \rho) \cap C$$
$$\text{and } T(W_{\lambda_0}, H_{\lambda_0}, \alpha, \beta) < T(W^\star, H^\star, \alpha, \beta)$$

This is a contradiction and we conclude that $\kappa = 1$.

This last proposition shows that the ratio of α and β is useful to specify the ratio of the norms of W and H at any local or global minimum. If $\alpha = \beta$, the norms will be equal when reaching a minimum.

We conclude from these propositions that for a given number of profiles k and a positive regularization parameter α solving the NMF problem is reduced to finding $(W^\star, H^\star) \in C$ such that

$$(W^\star, H^\star) = \underset{(W,H)\in C}{\operatorname{argmin}} V(W, H, \alpha).$$

where $V(W, H, \alpha) = T(W, H, \alpha, \alpha)$.

As constrained optimization algorithms detect stationary points with no guarantee that they are local or global minima, we finish with the following proposition.

Proposition 4 *For all $\alpha, \beta > 0$, let $\boldsymbol{a} = (\beta/\alpha)^{1/4}$, then if $(W^\star, H^\star)$ is a stationary point of* (1) *in the Karush- Kuhn-Tucker (KKT) sense then $(\boldsymbol{a}W^\star, H^\star/\boldsymbol{a})$ is a stationary point of* (2).

Proof Let $(W^\star, H^\star) \in C$ be a stationary point for problem (1). Then, recalling the KKT conditions, there exist $U_H \in \mathcal{M}_{k,r}(\mathbb{R}^+)$, $U_F \in \mathcal{M}_{c,r}(\mathbb{R}^+)$ where c is the number of rows of F_Δ, and $U_W \in \mathcal{M}_{n,k}(\mathbb{R}^+)$ such that

$$\nabla_H \mathcal{L}(W^\star, H^\star, U_F, U_H, U_W, \alpha, \beta) = 0 \tag{5}$$

$$\nabla_W \mathcal{L}(W^\star, H^\star, U_F, U_H, U_W, \alpha, \beta) = 0 \tag{6}$$

$$U_H \circ H^\star = 0, \quad U_F \circ F_\Delta H^{\star T} = 0, \quad U_W \circ W^\star = 0, \tag{7}$$

where $\circ$ is the term to term product of matrices and the Langrangian $\mathcal{L}$ is

$$\mathcal{L}(W, H, U, V, S, \alpha, \beta) = T(W, H, \alpha, \beta) + tr(U^T F_\Delta H^T) - tr(V^T H) - tr(S^T W).$$

Let us define, for any matrix M, $\phi_a(M) = aM$. From Proposition 1, we obviously have for all W and H

$$\begin{aligned}&\mathcal{L}(W, H, U, V, S, \alpha, \beta) = \\ &\quad \mathcal{L}(\phi_a(W), \phi_{1/a}(H), \phi_a(U), \phi_a(V), \phi_{1/a}(S), \sqrt{\alpha\beta}, \sqrt{\alpha\beta}),\end{aligned}$$

and from the chain rule

$$\begin{aligned}
&\nabla_H \mathcal{L}(W^\star, H^\star, U_F, U_H, U_W, \alpha, \beta) = \\
&\nabla_H \phi_{1/a}(H^\star) \nabla_{\phi_{1/a}(H)} \mathcal{L}(\phi_a(W^\star), \phi_{1/a}(H^\star), \phi_a(U_F), \phi_a(U_H), \phi_{1/a}(U_W), \sqrt{\alpha\beta}, \sqrt{\alpha\beta}) \\
&= \frac{1}{a} \nabla_{\phi_{1/a}(H)} \mathcal{L}(\phi_a(W^\star), \phi_{1/a}(H^\star), \phi_a(U_F), \phi_a(U_H), \phi_{1/a}(U_W), \sqrt{\alpha\beta}, \sqrt{\alpha\beta}), \\
&\nabla_W \mathcal{L}(W^\star, H^\star, U_F, U_H, U_W, \alpha, \beta) = \\
&\nabla_W \phi_a(W^\star) \nabla_{\phi_a(W)} \mathcal{L}(\phi_a(W^\star), \phi_{1/a}(H^\star), \phi_a(U_F), \phi_a(U_H), \phi_{1/a}(U_W), \sqrt{\alpha\beta}, \sqrt{\alpha\beta}) \\
&= a \nabla_{\phi_a(W)} \mathcal{L}(\phi_a(W^\star), \phi_{1/a}(H^\star), \phi_a(U_F), \phi_a(U_H), \phi_{1/a}(U_W), \sqrt{\alpha\beta}, \sqrt{\alpha\beta}).
\end{aligned}$$

So, obviously, $(\phi_a(W^\star), \phi_{1/a}(H^\star)) \in C$ is a stationary point of problem (2) whose Lagrangian is

$$\mathcal{L}(W, H, U, V, S, \sqrt{\alpha\beta}, \sqrt{\alpha\beta}),$$

and $\phi_a(U_F)$,$\phi_a(U_H)$ and $\phi_{1/a}(U_W)$ are non-negative matrices since they satisfy

$$\nabla_H \mathcal{L}(\phi_a(W^\star), \phi_{1/a}(H^\star), \phi_a(U_F), \phi_a(U_H), \phi_{1/a}(U_W), \sqrt{\alpha\beta}, \sqrt{\alpha\beta}) = 0$$

$$\nabla_W \mathcal{L}(\phi_a(W^\star), \phi_{1/a}(H^\star), \phi_a(U_F), \phi_a(U_H), \phi_{1/a}(U_W), \sqrt{\alpha\beta}, \sqrt{\alpha\beta}) = 0$$

$$\phi_a(U_H) \circ \phi_{1/a}(H^\star) = 0, \quad \phi_a(U_F) \circ F_\Delta \phi_{1/a}(H^{\star T}) = 0, \quad \phi_{1/a}(U_W) \circ \phi_a(W^\star) = 0.$$

Conversely, if $(W'^\star, H'^\star) \in C$ is a stationary point of problem (2), then $(\phi_{1/a}(W'^\star), \phi_a(H'^\star)) \in C$ is a stationary point of problem (1).
