# Supplementary material for: Inferring Aggregated Functional Traits from Metagenomic Data Using Constrained Non-negative Matrix Factorization: Application to Fiber Degradation in the Human Gut Microbiota
Source: PLoS Comput Biol. 2016 Dec 16;12(12):e1005252. doi: 10.1371/journal.pcbi.1005252 (PMC5161307; doi:10.1371/journal.pcbi.1005252)
Supplement: S2 Text — This file provides details about optimization steps (21) and (22). (PDF) [file pcbi.1005252.s002.pdf]

Nesterov's first order method applied to NMF

Nesterov's algorithm. As in [1], we use Nesterov's first order method (see [2] and [3]) in each of the minimization step. This accelerated projected gradient method allows solving convex non-smooth optimization problems with guaranteed quadratic convergence rate. From [3], it applies to problems of the form:

$$x^\star = \underset{x}{\operatorname{argmin}}\; f(x) + P(x), \tag{1}$$

where $f(x)$ and $P(x)$ are proper lower semi continuous and convex, $P(x)$ is defined on a closed set named $domP$, $f(x)$ is defined on an open set containing $domP$, the gradient of f, $\nabla f(x)$ exists and is Lipschitz continuous on $domP$. Let x^0 be the initial guess for the minimum, and L be the Lipschitz constant associated with $\nabla f(x)$, which coincides with the largest singular value of the Hessian of $f(x)$ if f is twice differentiable. The algorithm is the following.

Algorithm 1 Nesterov Step

$S^{(0)} \leftarrow x^0$
$Z^{(0)} \leftarrow x^0$
$\theta^{(0)} \leftarrow 1$
while Convergence $\neq$ True **do**

$$\begin{aligned} Y^{(k)} &= (1-\theta^{(k)})S^{(k)} + \theta^{(k)} Z^{(k)} \\ Z^{(k+1)} &= \operatorname{Prox}_{P/(L\theta^k)}\left[Z^{(k)} - \frac{1}{L\theta^{(k)}}\nabla f(Y^{(k)})\right] \\ S^{(k+1)} &= (1-\theta^{(k)})S^{(k)} + \theta^{(k)} Z^{(k+1)} \\ \theta^{(k+1)} &= \frac{1}{2}\theta^{(k)}(\sqrt{(\theta^{(k)})^2+4} - \theta^{(k)}) \end{aligned} \tag{2}$$

end while
$x^\star \leftarrow S^{k+1}$

Equation (2) refers to the proximal operator of function $P(x)/(L\theta^{(k)})$, expressed as

$$\text{Prox}_{P/(L\theta^k)}[v] = \underset{u}{\text{argmin}} \frac{P(u)}{L\theta^k} + \frac{1}{2}\|u - v\|_F^2. \tag{3}$$

Application to NMF inference problem. From now on, we consider the normalized matrices and drop the tilde notation for A and H and set

$$V(W, H, \alpha) = \|A - WH\|_F^2 + \alpha(\|\mathbf{1}^t H\|_2^2 + \|W\|_F^2).$$

The inference procedure consists in alternate optimisation steps on W and H.

Optimisation step on W :

$$W^{(t+1)} \leftarrow \underset{\boldsymbol{W} \geq 0}{\text{argmin}} \quad \|A - \boldsymbol{W}H^{(t)}\|_F^2 + \beta\|\boldsymbol{W}\|_F^2$$

This step is solved using Nesterov's algorithm where P is defined as the positive indicator function for matrices

$$P(\boldsymbol{W}) = \begin{cases} 0 \text{ if } \boldsymbol{W} \geq 0 \\ +\infty \text{ otherwise.} \end{cases} \tag{4}$$

Moreover, we set $f(\boldsymbol{W}) = V(\boldsymbol{W}, H^{(t)}, \alpha)$. An easy computation shows that $\text{Prox}_{P/(L\theta^k)}(v)$ is the euclidean projection onto non negative numbers:

$$\text{Prox}_{P/(L\theta^k)}[v_{i,j}] = \begin{cases} v_{i,j} \text{ if } v_{i,j} \geq 0 \\ 0 \text{ else} \end{cases} \tag{5}$$

A straightforward computation provides the expression of the gradient term in (2) as

$$\nabla_{\boldsymbol{W}} V(\boldsymbol{W}, H, \alpha) = 2\boldsymbol{W}(HH^t + \alpha \; Id) - 2AH^t,$$

where Id denotes the $k \times k$ identity matrix.

Optimization step on H :

$$H^{(t+1)} \leftarrow \underset{\boldsymbol{H} \geq 0, F_{\triangle}\boldsymbol{H}^t \leq 0}{\text{argmin}} \quad \|A - W^{(t+1)}\boldsymbol{H}\|_F^2 + \alpha\|\mathbf{1}^t\boldsymbol{H}\|_2^2$$

In this case, we cannot directly use Nesterov's algorithm, since there is no straightforward expression for the proximal operator (projection on the constraints set). Instead, we use a standard Augmented Lagrangian Method [4], combining Uzawa iterations with Nesterov's first order method. We define

$$\mathcal{L}(H,U) = V(W^{(t+1)}, \boldsymbol{H}, \alpha) + tr(\boldsymbol{H}^t \boldsymbol{U}^t \bar{F})$$

where $tr()$ is the trace operator and U is the $c \times k$ matrix of the Lagrange multipliers, with non negative entries. Moreover, $[.]_+$ is the euclidean projection on non negative numbers, a is the smallest eigenvalue of the Hessian matrix of $\mathcal{L}$ as a function of H, and L is the module of the largest eigenvalue of F_Δ. Then we use Algorithm 2, in which problem (6) is solved using 1.

Algorithm 2 Uzawa step

while Convergence $\neq$ True **do**

$$H^{(k+1)} = \underset{\mathbf{H}\geq 0}{\text{argmin}} \quad \mathcal{L}(\mathbf{H}, U^{(k)}) \tag{6}$$

$$U^{(k+1)} = \left[U^{(k)}) + \frac{2a}{L^2} F H^{(k+1)T} \right]_+$$

end while

References

1. Naiyang Guan, Dacheng Tao, Zhigang Luo, and Bo Yuan. NeNMF: An optimal gradient method for nonnegative matrix factorization. *IEEE Transactions on Signal Processing*, 60(6):2882–2898, 2012.

2. Yu Nesterov. *Introductory Lectures on Convex Optimization: a Basic course.* Springer US, 2004.

3. P Tseng. On accelerated proximal gradient methods for convex-concave optimization. *submitted to SIAM Journal on Optimization*, 2008.

4. K. J. Arrow, L. Hurwicz, and H. Uzawa. *Studies in linear and non-linear programming.* Stanford mathematical studies in the social sciences. Stanford University Press, 1958.
