# Supplementary material for: Inferring Aggregated Functional Traits from Metagenomic Data Using Constrained Non-negative Matrix Factorization: Application to Fiber Degradation in the Human Gut Microbiota
Source: PLoS Comput Biol. 2016 Dec 16;12(12):e1005252. doi: 10.1371/journal.pcbi.1005252 (PMC5161307; doi:10.1371/journal.pcbi.1005252)
Supplement: S3 Text — This file details the selection of the regularization parameter α based on the three criteria. (PDF) [file pcbi.1005252.s003.pdf]

Selection of the regularisation parameter α

Figures 1 and 2 display the bi-cross validation error, the reconstruction error, and the concordance of H for various values of k. The three criteria are globally constant when α is smaller that $10^{-1.5} = 0.0316$ and deteriorate more significantly when $\alpha = 0.1$ for most values of k, except for the concordance of H for $k = 6$, which displays a constant decrease rate as α increases; nevertheless, the distribution of the concordance for each random split indicates that the variations of the mean are not strongly significant. Therefore, the value $\alpha = 10^{-1.5}$ was chosen.

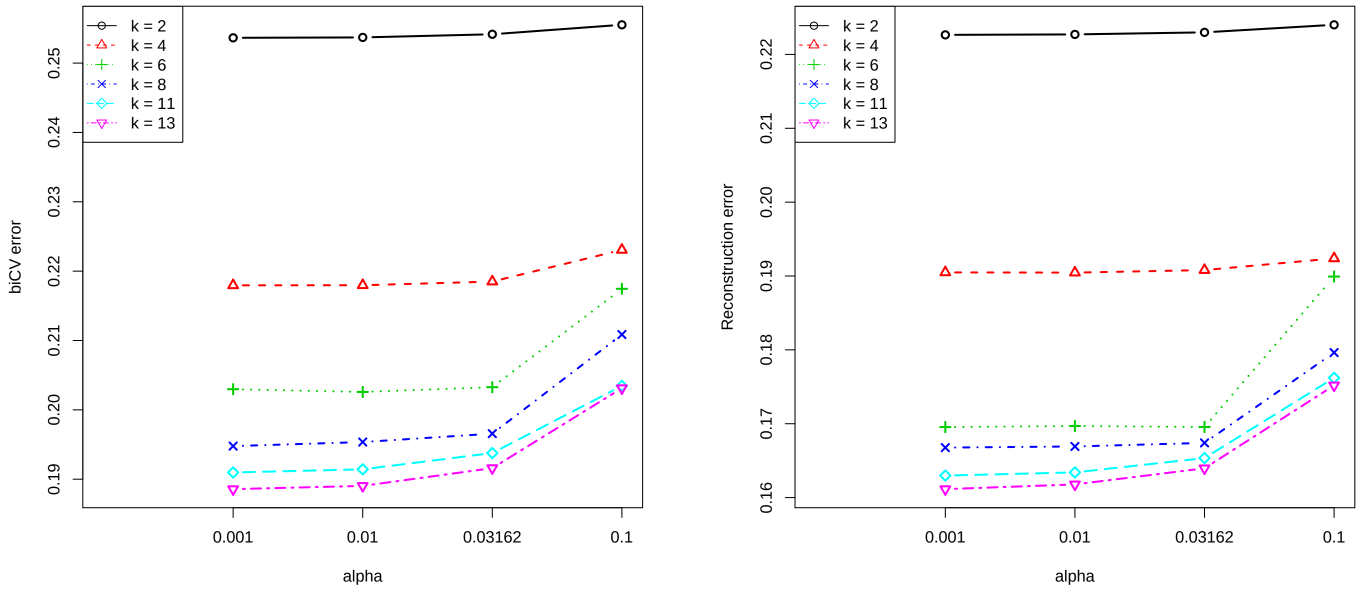

Figure 1: Bi-cross validation error (left) and reconstruction error (right) as a function of α, for various values of k

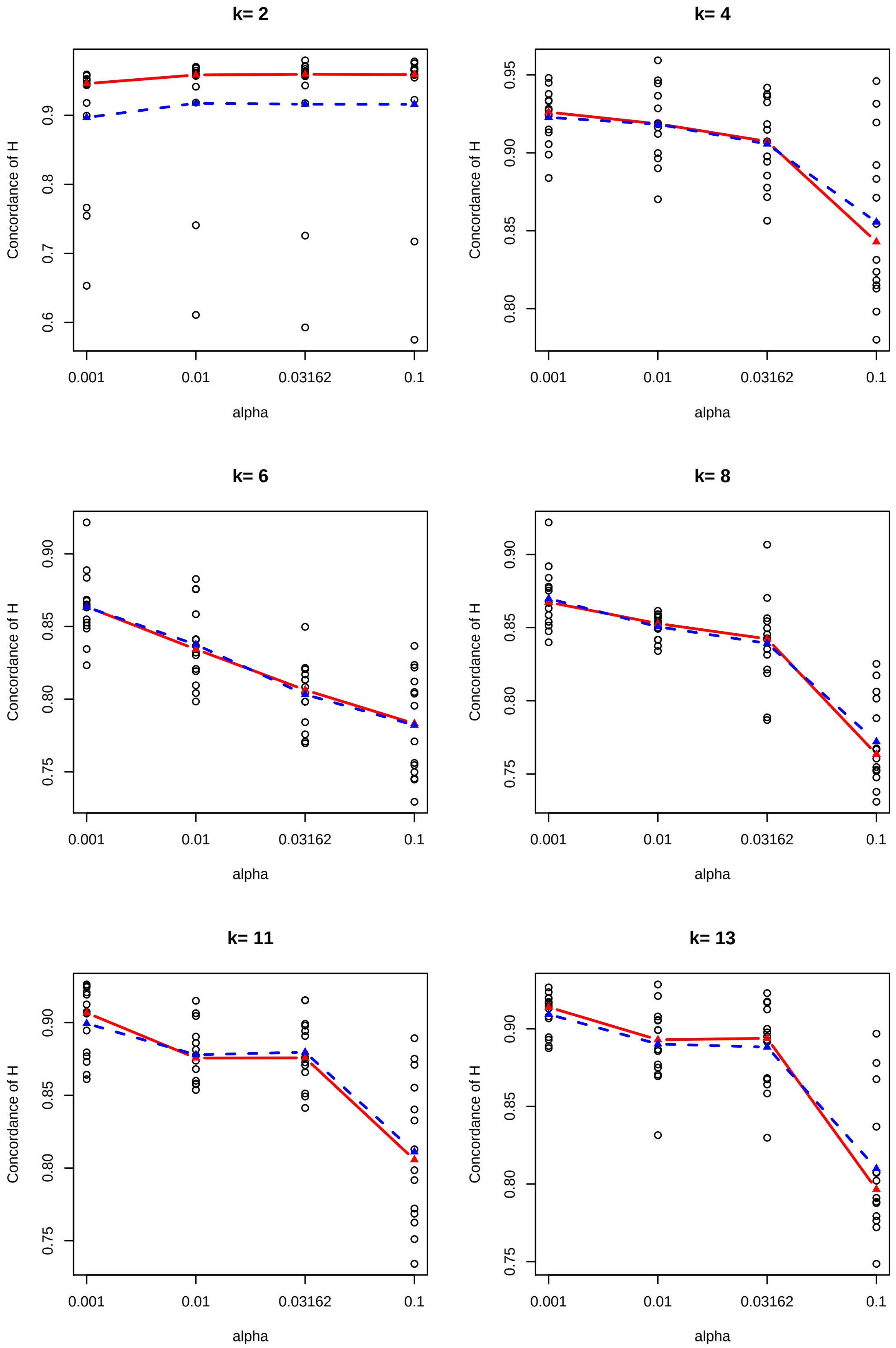

Figure 2: Concordance of H for each random split (black circles), median (dotted blue line) and mean (solid red line) as a function of α, for various values of k
